# Animal biodiversity and specificity in children’s picture books

**DOI:** 10.1177/09636625221089811

**Published:** 2022-05-09

**Authors:** Michiel Jan Dirk Hooykaas, Marloes Gertrudis Holierhoek, Joris Sebastiaan Westerveld, Menno Schilthuizen, Ionica Smeets

**Affiliations:** Leiden University, The Netherlands; Leiden University, The Netherlands; Naturalis Biodiversity Center, The Netherlands; Leiden University, The Netherlands

**Keywords:** anthropomorphism, biodiversity, cultural representations, picture books, taxonomic bias, specificity

## Abstract

While animal biodiversity is declining globally, cultural representations of animals are highly prevalent in society and play an increasing part in shaping children’s perceptions of animal diversity. We studied animal portrayals in children’s picture books in the Netherlands, and coded over 2,200 animals from 217 award-winning books. We found a strong bias toward vertebrates, mammals in particular. Mammals were featured more often than other animals, played more prominent roles in the story, and were visually and textually specified more strongly. Furthermore, exotic and domestic species outnumbered native species. Picture books currently are likely to reinforce children’s perceptions toward only a small part of animal biodiversity. While we realize that picture books have other primary aims, picture book makers could be inspired and encouraged to diversify and specify their portrayals of the natural world. This would broaden children’s perceptions of the animal kingdom and could help foster lasting connections to biodiversity.

## 1. Introduction

Animal biodiversity is declining worldwide, with a large impact on humans and non-human animals alike (Ceballos et al., 2017; Dirzo et al., 2014). As conservation relies on public support (Home et al., 2009) and people tend to care about what they know (Schlegel and Rupf, 2010; Wilson and Tisdell, 2005), awareness in society about animals and their diversity is imperative. However, studies have demonstrated that people in Western societies have limited knowledge about animals; for example, perceptions seem to be directed mostly toward exotic and domestic species, notably mammals (Ballouard et al., 2011; Lindemann-Matthies, 2005). It has been hypothesized that this lack of awareness is caused by a widening gap between humans and nature (Miller, 2005). Authors have noted that especially in highly urbanized countries, people have less opportunity and less motivation to experience biodiversity outdoors, resulting in an “extinction of experience” (Cox et al., 2017; Pyle, 2011; Soga and Gaston, 2016). This may prevent people from learning about animals and developing meaningful connections with them.

However, people may also encounter animals indirectly, when they are exposed to cultural products that portray animals, such as books and films. These cultural representations can be regarded as agents of socialization that help build and reinforce perceptions (Gerbner, 1969; Kesebir and Kesebir, 2017; Potter, 2014). For instance, it has been reported that animal portrayals can foster knowledge about species (Pearson et al., 2011), raise interest (Fernández-Bellon and Kane, 2019; Fukano et al., 2020; Soga et al., 2016), and trigger feelings of empathy (Kalof et al., 2015; Pearson et al., 2011). Products aimed at children play a particularly important role, as young children are sensitive to cultural discourse about animals (DeMello, 2012; McCrindle and Odendaal, 1994), and their knowledge levels and affinities toward animals affect their future perceptions and pro-conservation behaviors (Hinds and Sparks, 2008; Kahn, 2002; Kellert, 1985, 2002; Pilgrim et al., 2007), making it important to understand the image that cultural products targeted at children convey of animals.

### The potential of picture books

One product that features animals and that possibly impacts children’s perceptions of animal diversity is a picture book. Most children in Western societies are exposed to picture books (Ghonem-Woets, 2009; Van den Eijnden, 2015), and while picture book makers rarely depict animals to transfer factual information about them, they do portray animals in their stories and artwork. Animals may be portrayed as minor characters that illustrate environment settings, but they may also feature prominently as main and supporting characters, for example, to serve as human replacements for comical purposes or to teach moral lessons and appropriate social behavior (Larsen et al., 2017; Sousa et al., 2017).

Picture books thus expose young readers subtly and repeatedly to animals, and in line with cultivation theory (Gerbner, 1969; Potter, 2014) and research on the impact of subtle, repeated exposure (Bornstein and D’Agostino, 1992; Kaikati and Kaikati, 2004; Roy and Chattopadhyay, 2010; Zajonc, 1968) they are likely to shape children’s perceptions of animal diversity and their feelings about animals (Prokop et al., 2011; Root-Bernstein et al., 2013). For instance, children may learn to distinguish and name different animals and may grow affinity toward animals that play leading roles in compelling stories. Previous studies have already demonstrated that young children are able to learn new biological facts from realistic picture books (Ganea et al., 2011; Kelemen et al., 2014). Picture books have further been used purposefully to expand children’s vocabulary (Larragueta and Ceballos-Viro, 2018; Sénéchal et al., 1996) and visual literacy (Read and Smith, 1982), and to teach various subjects, ranging from environmental protection (Hsiao and Shih, 2016) and mathematics (Van den Heuvel-Panhuizen et al., 2009) to healthy foods (Heath et al., 2014) and ethnic and gender diversity (Wissman, 2019). In line with this, educators may wish to use picture books to introduce children to the animal kingdom, to help to counterbalance the loss of direct experiences with animals in nature.

However, several factors may limit children’s opportunities to learn about animals through picture books. First, authors and illustrators may restrict their portrayals to a small number of well-known animals. It has been reported for different cultural products that mammals predominate (Fernández-Bellon and Kane, 2019; Huxham et al., 2006; Nemésio et al., 2013), and that exotic and domestic species outnumber native, wild species (Celis-Diez et al., 2016; Genovart et al., 2013; Moreno-Tarín et al., 2021). Skewed portrayals could explain why children’s perceptions currently seem to be directed mostly toward these animals (Ballouard et al., 2011; Genovart et al., 2013; Lindemann-Matthies, 2005). For instance, it has been shown that children are unaware of many common animal species, that is, there is a high “species illiteracy” (Hooykaas et al., 2019). Biases could also explain misconceptions about species richness and abundance (Courchamp et al., 2018; Platt, 2019; Vázquez-Plass and Wunderle, 2010).

Second, children’s opportunities to learn may be compromised by low specificity of portrayals. Artistic work can be highly distorted from reality (Marriott, 2002), and as a result depictions of animals may be identified only at a higher taxonomic level (e.g. as “an insect”), offering little room to foster species literacy. Even when depictions are realistic or iconic, animals may still not be identified correctly if text references are lacking or are unspecified (e.g. when a blackbird is referred to as “bird”).

Finally, picture book makers may portray animals anthropomorphically, for example, with clothes or accessories, human behavior, or human facial expressions. This may make them relatable and likable for children (Chan, 2012; Geerdts, 2016; Root-Bernstein et al., 2013), and some argue that subtle anthropomorphism in children’s storybooks can aid in the learning of biological facts (Geerdts et al., 2016a; McCabe and Nekaris, 2018). However, others have noted that anthropomorphization can negatively affect children’s knowledge of animals (Ganea et al., 2014; Geerdts et al., 2016b; Marriott, 2002; Waxman et al., 2014); for instance, it may limit recognizability and may induce misconceptions, as it can be challenging for children to differentiate what is real from what is true only in the story world (Strouse et al., 2018).

### Aim of this study

As the human population grows and urbanization continues, cultural representations will increasingly mediate people’s interactions with animals (Kellert, 2002), showing the importance of understanding what picture they convey. Picture books have been researched in the past for their representation of ethnic diversity and gender with the underlying idea that diverse portrayals can help develop an inclusive worldview (De Bruijn et al., 2021; Harlin and Morgan, 2009; Trepanier-Street and Romatowski, 1999). With a similar approach in mind, we aimed to elucidate the image of animals that picture books present to children, to clarify how the animal kingdom currently is appropriated in Western society and to explore learning opportunities for children.

We examined the portrayal of animals in picture books in the Netherlands, a highly urbanized country in Western Europe where species literacy of primary school children was found to be very low (Hooykaas et al., 2019). In societies with high levels of urbanization, indirect experiences play a significant part in shaping people’s perceptions of biodiversity (Prévot-Julliard et al., 2015; Soga et al., 2016), which makes it apt to study Dutch children’s books. Whereas previous studies have investigated animal portrayals in children’s books recommended for usage in classrooms (Celis-Diez et al., 2016; Sousa et al., 2017), we examined award-winning picture books, as these are generally sold well (Squires, 2007) and are often read in non-school settings. Moreover, while other studies have mentioned children’s books as a small part of a broader study (Genovart et al., 2013; Huxham et al., 2006), we studied in depth the diversity, specificity, and anthropomorphization of animals in different roles.

To determine the diversity of animals represented in picture books and the way in which they are portrayed, we established which animal species, families, orders, and classes were most prevalent, analyzed the specificity of depictions and textual references, and calculated the proportion of anthropomorphic animals. As animals can be accorded different roles in the stories in which they figure, we finally examined possible differences in taxonomic prevalence, specificity, and anthropomorphism between main, supporting, and minor characters.

We studied the following research questions:

1. Which taxa and types of animals (i.e. exotic or native, and domestic or non-domestic) are portrayed, and how does this differ between main, supporting, and minor characters?
2. To which taxonomic level are the animals specified in the imaging and text, and how does this differ between classes and between main, supporting, and minor characters?
3. What proportion of the portrayed animals are anthropomorphized, and how does this differ between classes and between main, supporting, and minor characters?

## 2. Methods

To capture the current representation of animals in picture books available to Dutch children, we performed a quantitative content analysis, as follows.

### Book selection

We included all books targeted at children aged 2–9 years that received an award in the Netherlands between 2010 and 2020 for best book, story, or artwork (Supplemental Appendix A). We excluded non-story books (e.g. seek and find books), omnibus editions, and books without illustrations to support the story. This yielded 217 book titles from 160 authors and 144 illustrators. The sample comprised 120 original Dutch books and 97 international books translated into Dutch.

### Sampling animals

We included depictions of both extant and extinct animals, as well as cultural representations of these animals (e.g. depicted teddy bears). However, we excluded fantasy animals (mythical creatures such as unicorns and dragons) and biodiversity elements such as feathers, footprints, and bones.

Per book we included all main characters (playing the leading role and serving as protagonists), supplemented by up to 20 other animals. The latter group could be supporting characters (playing a supporting role essential to the storyline) or minor characters (part of the scenery). Each animal species was included once for each role in which it figured (e.g. if the protagonist was a cow, and a herd of cows was visible in the background, “cow” was inserted twice, both as main and minor character). Animals mentioned in book titles were finally added to the sample if they had not already been coded; these could serve different roles in the storyline.

We started our selection on the first page of each story (e.g. skipping the cover), scanned each page from left to right and per page included the first five animals encountered. We avoided a scan from top to bottom, as this would have skewed results to flying animals, and we included a maximum per page to ensure covering different parts of the story. The animals included in the data set were photographed, so that codings could be checked when a book borrowed from the library had been returned.

### Coding animals

We constructed a codebook (Supplemental Appendix B) to code the sampled animals. Each animal was identified at the lowest possible taxonomic level, using context and cues (e.g. depicted scenery and text). Subsequently, the taxonomic affiliation was noted using the English Wikipedia (species, family, order, and class, and whether the animal was an invertebrate or vertebrate). For the purpose of this study, we treated dinosaurs as a taxonomic class, to separate them from other reptiles and birds. In addition, we coded the type of animal (native or exotic, domestic or non-domestic), using lists of animal species native to the Netherlands and a list of domestic animals (Supplemental Appendix C).

**Figure 1.**
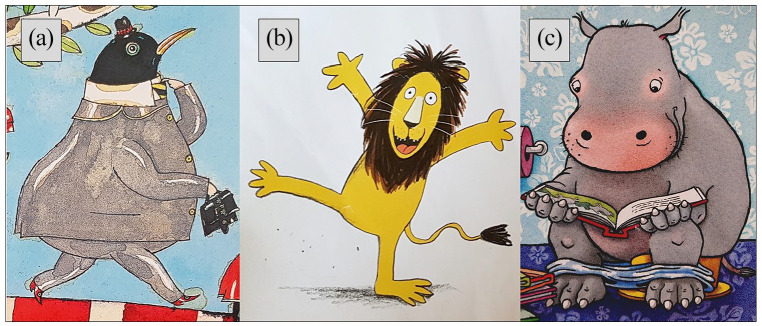
Different forms of anthropomorphism. Wearing clothing: a blackbird wearing a suit, hat, and briefcase (Houkema, 2010) (a); human facial expressions: a happy lion (Douglas and Riphagen, 2016) (b); human behavior: a hippopotamus reading on the toilet (Pfister, 2008) (c).

To explore recognizability of the animals and the level of distortion in the portrayal, we finally noted for each animal the lowest taxonomic rank at which it was mentioned in the text, the lowest taxonomic rank at which it could be identified, and the depiction state (visually anthropomorphized or not). We distinguished different types of anthropomorphism: wearing clothes or accessories (e.g. jewelry), human behavior (including speech, use of human objects, bipedal walk, and human posture), and human facial features (including facial expressions, blushing cheeks, and feminine eyelashes) (see Figure 1).

For each animal, depictions throughout the book were used for coding; for example, when an animal got dressed later in the story, it was coded as wearing clothes.

### Intercoder reliability

Coding was executed by three researchers. The lead author, who is well-versed in the subject of biodiversity, verified the species identifications and if needed consulted experts (e.g. a paleontologist to help identify dinosaur species). The role in which an animal figured and the three types of anthropomorphism were coded independently by the first two authors and intercoder reliability was assessed by comparing codes of a randomly chosen 10% of the animals. The level of agreement was strong (McHugh, 2012) for role (percent agreement = 91.5%, Cohen’s Kappa = 0.82), clothing/accessories (percent agreement = 98.7%, Cohen’s Kappa = 0.94), human behavior (percent agreement = 93.0%, Cohen’s Kappa = 0.82), and human facial features (percent agreement = 91.2%, Cohen’s Kappa = 0.80). The cases where there had been disagreement were resolved through discussion, after which the lead author double-checked similar cases elsewhere.

### Data analysis

We compiled the data in Microsoft Excel 365 and performed descriptive and statistical analysis in IBM SPSS Statistics 25.0. Using frequency tables, we first explored prevalence of taxonomic groups per role and in total. Subsequently, we used two-tailed chi-square tests of independence with a significance level of *p* ≤ .05 to analyze relationships between the categorical variables (taxonomic classes, role, anthropomorphism, and specificity of identification and text references). To account for multiple testing, we applied a strict Bonferroni adjustment when making multiple comparisons.

## 3. Results

Most books (97.3%) featured one or more animals, and in a majority (79.3%) animals were essential to the storyline, serving as main or supporting characters. The final data set (Supplemental Appendix D) comprised 2237 animals in total: 155 main characters, 544 supporting characters, and 1538 minor characters.

### Taxonomic diversity

The majority (85.5%) of the animals portrayed in the picture books represented vertebrates. Mammals (43.9%) and birds (27.6%) were the most featured classes, followed by insects (9.8%), bony fish (5.7%), reptiles (4.0%), dinosaurs (2.6%), and amphibians (1.3%). Other taxonomic classes, whether vertebrate or invertebrate, were present in the data set only a few times or were lacking altogether (see Table 1).

**Table 1.** Prevalence of animal classes portrayed in children’s picture books (frequency counts for main, supporting, and minor characters, and total).

Class (ordered according to frequency)		Main	Supp.	Minor	Total	
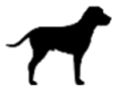	Mammals	111	297	575	983	43.9%
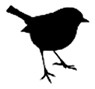	Birds	22	138	457	617	27.6%
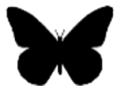	Insects	7	36	177	220	9.8%
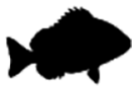	Bony fish	2	9	117	128	5.7%
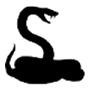	Reptiles	7	22	61	90	4.0%
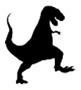	Dinosaurs	0	12	47	59	2.6%
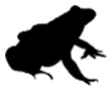	Amphibians	2	10	18	30	1.3%
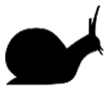	Snails and slugs	0	3	23	26	1.2%
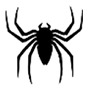	Arachnids	1	8	7	16	0.7%
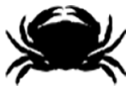	Crustaceans	1	2	10	13	0.6%
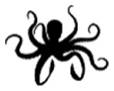	Cephalopods	2	2	6	10	0.4%
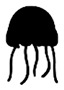	Jellyfish	0	1	8	9	0.4%
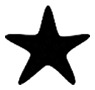	Echinoderms	0	0	9	9	0.4%
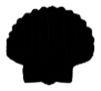	Bivalves	0	0	7	7	0.3%
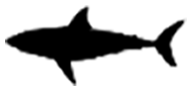	Cartilaginous fish	0	0	5	5	0.2%
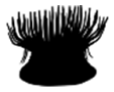	Sea anemones and corals	0	0	1	1	0.0%
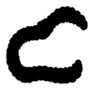	“Other invertebrates”	0	3	9	12	0.5%
	Total	155	544	1538	2237	100.0%

From the animals, 79.7% could be assigned to a taxonomic order and 65.5% to a taxonomic family. The animals represented 79 orders and 143 families, yet only a few were portrayed frequently (see Table 2 and Supplemental Appendix E).

**Table 2.** Top 20 most featured animal families portrayed in children’s picture books (frequency counts for main, supporting, and minor characters, and total).

Family (ordered according to frequency)		Main	Supp.	Minor	Total	
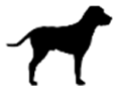	Canids; e.g. dog, fox	20	44	80	144	6.4%
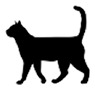	Felids; e.g. cat, lion	9	40	66	115	5.1%
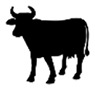	Bovids; e.g. cow, sheep	8	40	47	95	4.3%
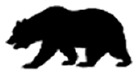	Bears; e.g. brown bear, polar bear	15	27	49	91	4.1%
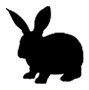	Rabbits and hares; e.g. rabbit, hare	14	19	51	84	3.8%
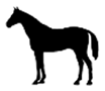	Horses; e.g. horse, donkey	4	19	52	75	3.4%
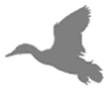	Ducks, geese, and swans; e.g. mallard, domestic goose	4	17	44	65	2.9%
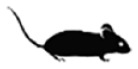	Mice; e.g. house mouse, rat	9	13	37	59	2.6%
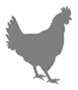	Phasianids; e.g. chicken, Indian peafowl	3	20	26	49	2.2%
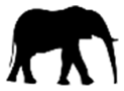	Elephants and mammoths; e.g. African elephant	9	17	22	48	2.1%
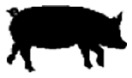	Pigs; e.g. pig, wild boar	4	18	24	46	2.1%
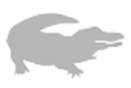	Crocodiles; e.g. crocodile	4	10	13	27	1.2%
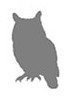	True owls; e.g. eagle-owl, snowy owl	1	12	14	27	1.2%
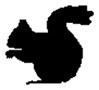	Squirrels; e.g. red squirrel	1	6	19	26	1.2%
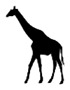	Giraffids; e.g. giraffe, okapi	4	6	15	25	1.1%
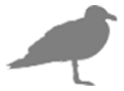	Gulls, terns and skimmers; e.g. gull	1	3	20	24	1.1%
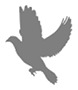	Pigeons and doves; e.g. rock pigeon	0	5	14	19	0.8%
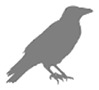	Corvids; e.g. crow, jackdaw	0	2	17	19	0.8%
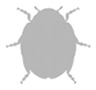	Ladybird beetles; e.g. seven-spot ladybird	3	1	14	18	0.8%
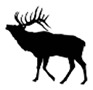	Deer; e.g. moose, reindeer	1	1	15	17	0.8%
	Other	41	224	899	1164	52.0%
	Total	155	544	1538	2237	100.0%

The animal icons in black, dark gray, and light gray represent mammals, birds, and other animals, respectively.

Carnivores were the most featured order, with a high number of canids, felids, and bears. Also numerous were “even-toed ungulates and cetaceans”, representing in particular bovids, pigs, and giraffids. Other mammalian orders that were portrayed often included rodents, due to the prevalence of mice and rats, odd-toed ungulates (mainly horses), rabbits and hares, and proboscideans, featuring mostly elephants (mostly elephants). Bird orders that were encountered frequently were songbirds and “waterfowl”: ducks, swans, and geese. In addition, gallinaceous birds (e.g. chicken), charadriiformes (e.g. gulls), and owls were quite common as well.

Considering insects, a considerable number of butterflies, beetles—represented frequently by ladybirds—and hymenopterans (mainly bees) were found. Reptilian orders that were featured frequently were squamates (e.g. snakes) and crocodilians (mostly true crocodiles), while Saurischian dinosaurs (e.g. theropods and sauropods) represented the most encountered order of dinosaurs. Amphibians were represented predominantly by frogs.

Only 39.4% of the animals could be identified as distinct species. The top 20 comprised mostly mammals, especially domestic animals (e.g. dog, cat, horse), supplemented by a few native (e.g. red fox, wolf, red squirrel) and exotic species (e.g. brown bear, lion, hippopotamus)—see Supplemental Appendix E. In total, 155 different animal species were encountered.

**Figure 2.**
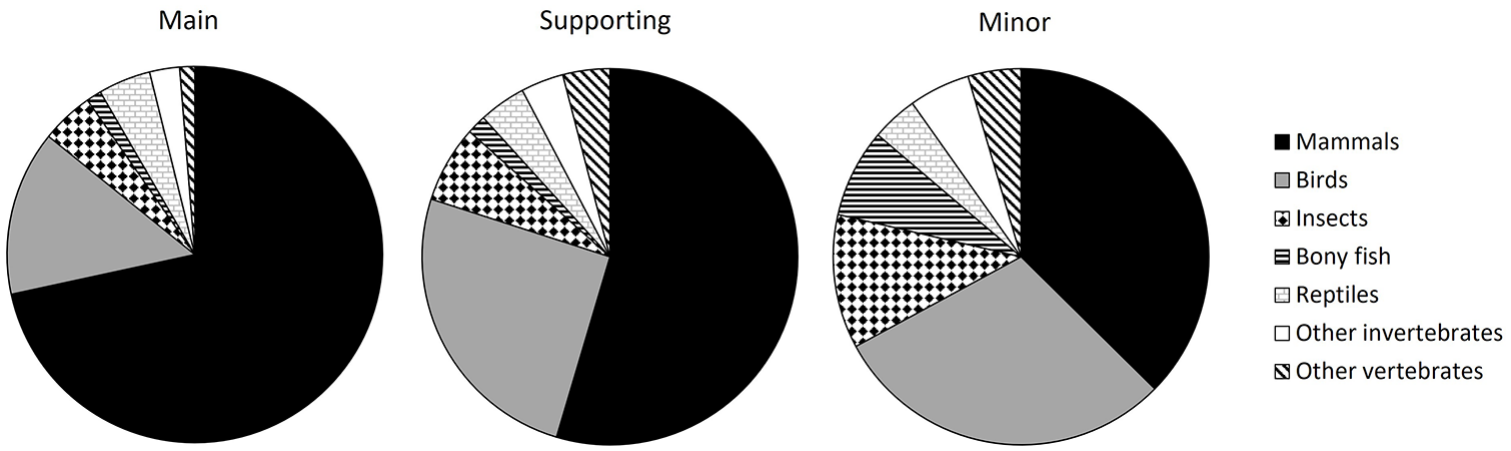
Proportion of animals featured in children’s picture books belonging to a particular class, for main, supporting, and minor characters.

The most abundant species, families, orders, and classes were similar in distribution among main, supporting, and minor roles (see Tables 1 and 2, Supplemental Appendix E). However, even though mammals were consistently the top featured class, they were particularly dominant in the leading role, while birds, insects, and bony fish were more prevalent as minor characters (see Figure 2).

### Type of animals

Virtually all animals (97.3%) represented extant animals. A quarter of these (24.3%) were domestic and represented companion (e.g. cat, dog) or farm animals (e.g. horse, pig). In fact, of the top ten most featured animal species, nine were domestic. Main and supporting characters were more likely to represent domestic species than minor characters (χ^2^(2) = 36.164, *p* < .001, Cramér’s *V* = 0.134).

Of the extant animals for which the origin could be determined, two-thirds (65.6%) were exotic (e.g. bear, crocodile, penguin) and one-third (34.4%) were native (e.g. common blackbird, mallard, red fox). Prevalence of exotic animals did not differ between roles, and books from Dutch publishers did not portray native animals more frequently than international publishers. Finally, we note that one in five animals (20.3%) was a cultural representation (e.g. cuddly toy, statue, or painting).

### Specificity of portrayals

Depending on their role in the story, animals were depicted prominently or inconspicuously. Often the depictions were abstracted, prototypical (e.g. generic birds), or unrealistic; for example, we noticed inaccuracies, such as a female blackbird character portrayed with male plumage. Whether an animal name was mentioned in the text depended on the role of the character in the story. While most of the main (78.7%) and supporting characters (82.7%) were mentioned in the text, minor characters were referred to only occasionally (16.7%).

The majority of text references were above species level (59.8%), yet there were differences between classes (χ^2^(8) = 190.401, *p* < .001, Cramér’s *V* = 0.479). Whereas the majority of references to mammals (61.3%) were at the species level, only 15.6% of references to other animals were species specific. Birds were frequently mentioned at the class (“bird”), or family level (e.g. “penguin”, “duck”, “woodpecker”), while references to reptiles and insects were generally at the order (e.g. “snake”, “turtle”, “butterfly”) or family level (e.g. “crocodile”, “bee”). Moreover, dinosaurs were mentioned mainly at the class level (“dinosaur”) and invertebrates other than insects were generally referred to at the class (e.g. “snail”) or order (e.g. “spider”) level. Bony fish were usually mentioned as “fish,” an informal name that may refer to animals from different classes (Supplemental Appendix F).

The greater specificity in references to mammals was found for both main (χ^2^(1) = 12.480, *p* < .001, Cramér’s *V* = 0.320), supporting (χ^2^(1) = 99.075, *p* < .001, Cramér’s *V* = 0.469), and minor characters (χ^2^(1) = 72.664, *p* < .001, Cramér’s *V* = 0.532). We checked whether the higher specificity in references to mammals stemmed from the abundance of domestic animals in the data set, which were often mammalian and mentioned at the species level more frequently than non-domestic species (χ^2^(1) = 291.675, *p* < .001, Cramér’s *V* = 0.618), but this was not the case.

The limited specificity of visual and textual portrayals affected recognizability, and overall only 39.4% of the animals could be identified as distinct species, many representing domestic animals. In addition, 4.9% of the animals were identified at the genus level (e.g. giraffe), 21.2% at the family level (e.g. ladybird), 14.2% at the order level (e.g. beetle), and 20.2% at the class level (e.g. insect). Mammals were recognizable as distinct species much more frequently (65.8%) than other animals (18.7%); (χ^2^(1) = 511.401, *p* < .001, Cramér’s *V* = 0.478). Furthermore, main and supporting characters were identified at the species level more frequently (56.1% and 53.1%) than minor animal characters (33.2%); (χ^2^(2) = 88.281, *p* < .001, Cramér’s *V* = 0.199).

### Anthropomorphism

Anthropomorphism was encountered in most books (77.4%); in total 42.1% of the animals were portrayed anthropomorphically. While the majority of the main (96.1%) and supporting characters (63.2%) were anthropomorphic, only 29.2% of the minor characters were accorded with human characteristics; the differences were significant (χ^2^(2) = 390.384, *p* < .001, Cramér’s *V* = 0.418). Human facial features were the most common way in which the animals were anthropomorphized (33.5%), followed by human behavior (25.9%), and clothing/accessories (14.3%); this pattern was found for main, supporting, and minor characters (Supplemental Appendix G). Animals were often anthropomorphized in multiple ways simultaneously.

Anthropomorphism differed between taxonomic classes. Mammals were anthropomorphized more frequently (57.3%) than other animals combined (30.2%) (see Table 3). They were depicted regularly with clothes or accessories, human behavior, and human facial features, while anthropomorphism was rare especially in portrayals of birds and fish. However, mammals were anthropomorphized more frequently only in the supporting (χ^2^(1) = 31.033, *p* < .001, Cramér’s *V* = 0.239) and minor role (χ^2^(1) = 66.097, *p* < .001, Cramér’s *V* = 0.207); no significant difference was found between mammals and other animals for main characters. Moreover, we note that amphibians and reptiles were accorded with human facial features and behavior relatively often too (Supplemental Appendix G).

**Table 3.** Comparison between the prevalence of different types of anthropomorphism in mammals and other animals.

Type of Anthropomorphism	Mammals	Other animals	χ^2^	ϕ_c_
Clothing	22.4% (220/983)	8.1% (101/1254)	92.020***	0.203
Behavior	37.3% (367/983)	16.9% (212/1254)	119.878***	0.231
Facial features	46.0% (452/983)	23.7% (297/1254)	123.011***	0.234
Any	57.3% (563/983)	30.2% (379/1254)	165.403***	0.272

χ^2^ = chi-square value; ϕ_c_ = effect size (phi coefficient or Cramér’s *V*).

Degrees of freedom was 1 for each comparison.

*** *p* < .001.

## 4. Discussion

Although most picture books are not specifically designed to educate children about the natural world, they may play an important role in offering children opportunities to learn about animals. We examined the image that picture books convey of animals and their diversity, by sampling animal portrayals from a large collection of prize winning picture books in the Netherlands.

### A skewed portrayal

Animals were abundant in our sample of award-winning children’s books, and they regularly played an essential role in the story. However, the portrayal was highly skewed toward vertebrates, particularly mammals, a pattern in line with previous research on picture books (Huxham et al., 2006; Sousa et al., 2017) and other cultural products aimed at children, such as magazines (Vrla et al., 2020). While mammals predominated, especially as main and supporting characters, other animals such as birds, insects, and bony fish were portrayed less frequently and often figured as minor characters, even though actual species richness and abundance is higher for these groups than for mammals. In fact, invertebrates account for over 95 percent of worldwide biodiversity (Brusca et al., 2016). Apart from taxonomic biases, exotic and domestic animals were highly abundant, in line with previous research on cultural representations (Ballouard et al., 2011; Celis-Diez et al., 2016; Huxham et al., 2006).

The biases that we found may be explained by a strategy of featuring animals that are generally loved and known by readers. By portraying mammals, particularly domestic species and charismatic, exotic animals such as bears and lions, picture book makers tap into people’s affinities for “loveable” animals with fur and forward-facing eyes (Albert et al., 2018; Genovart et al., 2013; Hooykaas et al., 2019; Lindemann-Matthies, 2005; Macdonald et al., 2015), while the limited presence of invertebrates, especially in essential roles, may flow from assumptions that these animals do not appeal to people (Batt, 2009; Kellert, 1993). However, the portrayal also partly reflects abundance and the actual likelihood of encountering animals. For instance, depicting insects and birds as background characters mirrors real experiences in nature, while the prevalence of domestic species and cultural representations (e.g. teddy bears) may be explained by the anthropogenic environments in which many stories were set. Such domestic settings may be easy to relate to for children growing up in Western societies. Finally, the biases are likely to stem partly from skewed perceptions of authors and illustrators (Kesebir and Kesebir, 2017), as they can only portray what they are aware of themselves.

The biases that we found may hinder children in developing an accurate understanding of animal diversity. For instance, children may assume that frequently depicted species are abundant even though they may occur in low numbers in the wild (Courchamp et al., 2018; Platt, 2019). A bias toward mammals in cultural products may further explain why children generally identify native mammals more readily than birds and insects (Hooykaas et al., 2019), even though outdoors they are more likely to encounter the latter. Moreover, as mostly exotic and domestic animals are featured, children may conclude that animals worthy of their attention can only be found abroad or in domestic settings (Ballouard et al., 2011; Lindemann-Matthies, 2005; Verboom et al., 2004). This is unfortunate, because native species can provide children with opportunities to develop a “sense of place,” a feeling of attachment to the local environment (Horwitz et al., 2001).

### Specificity and anthropomorphism

Many animals were portrayed in simplified and abstracted ways, and text references were often missing or above the species level. The visual distortions and the limited text references reduced recognizability, and overall only a minority of the animals could be identified as distinct species, the majority representing animals that are generally well known (e.g. domestic species). Specificity of the portrayals further differed between roles and taxa. Main and supporting characters were specified more than minor characters, and mammals were specified more than other taxa, who were regularly depicted as generic prototypes and mentioned at high taxonomic levels.

Whereas experts may accurately identify animals even when representations are distorted or when text references are missing, laypeople may not. Portrayals with low specificity will not help expand laypeople’s limited ability to distinguish and name species, which is unfortunate, as people tend to care about what they know (Balmford et al., 2002) and an inability to name parts of the natural world may lead to a loss of attention for it (Macfarlane, 2015, 2017). Since mammals were portrayed with higher specificity, picture book makers may further inadvertently create the impression that other animals are less diverse.

Many animals were further portrayed with human facial features, human behavior, or clothes. In many stories, animals acted as human substitutes; for example, they lived in a house and celebrated birthdays. Notably, main characters were anthropomorphized, probably because it is deemed to be most important for them to be relatable and likable for readers. Moreover, leading characters were usually featured prominently and frequently, making them relatively easy to anthropomorphize. Likewise, some animals, notably mammals, were anthropomorphized more frequently than others probably because they can be accorded human characteristics more easily; for example, bipedal walk is hard to include in portrayals of fish and snakes. However, certain types of anthropomorphism (e.g. human facial expressions) were common in animals other than mammals too, especially in amphibians and reptiles.

Anthropomorphization probably reduces recognizability by distorting the link with the real animal that a character represents, and may induce misconceptions (Ganea et al., 2014; Geerdts et al., 2016; Marriott, 2002; Waxman et al., 2014). For example, anthropomorphic non-conspecific animal characters in stories often help each other, whereas in reality cooperative behavior between different species is rare. Although friendly portrayals may trigger positive feelings and facilitate connections with animals (Chan, 2012; Geerdts, 2016; Root-Bernstein et al., 2013), they can also lead people to think that wild animals can be readily approached without risk (Barney et al., 2005; Root-Bernstein et al., 2013; Tate and Pelton, 1980). Compared with the comical and stereotypical characters in picture books, real animals may further appear dull (Oswald, 1995), and differences in anthropomorphization between taxa may lead children to view some animals as loveable subjects and other animals as mere objects (Cole and Stewart, 2016; Root-Bernstein et al., 2013).

### Directions for future research

It is important to emphasize that portrayals do not automatically translate to learning outcomes and changed attitudes. Children experience difficulty in differentiating reality and fantasy, and it is unlikely that children always link highly transformed figures to the real animals that they represent (Strouse et al., 2018). Even animals that are portrayed realistically may be difficult to identify, for example, when there are large shifts in perspective and an animal is depicted relatively small on one page, and large on another (Dove, 2011; Poulsen et al., 1979). However, even when children are not able to identify an animal accurately, they may still develop interest in animals and learn about them. For example, a story about an exotic caterpillar that transforms into a butterfly will teach a child about the lifecycle of native butterflies too. Further research is needed to determine the exact impact of animal portrayals in picture books on children.

Furthermore, the vital role of parents and teachers should not be overlooked, as by reading stories to children they play a vital part in mediating the exposure to animal biodiversity (Greenhoot et al., 2014; Justice et al., 2005). Depending on their own prior knowledge, parents and teachers will elaborate more or less about the animals that are depicted. Moreover, they may not be aware of suitable books and ways to use them; for example, opportunities to discuss with children (Duursma et al., 2008; Strouse et al., 2018). It is thus important to explore how teachers and parents can be encouraged and supported.

Finally, we note that knowledge about animals encompasses more than the ability to identify them. Apart from identification skills, species literacy also involves knowledge about species’ habitat, diet, and living community (e.g. what kind of animals naturally occur together). We noticed that animals were often displaced from their natural environment, as most stories took place in human-altered settings, and the few books that did portray natural landscapes usually displayed highly simplified habitats. This links to studies reporting misconceptions in children about the places where animals occur (Strommen, 1995; Torkar, 2016). Moreover, animals regularly ate human food and were portrayed alongside species that they would never encounter in the wild. Future studies on picture book representations could incorporate such dimensions of biodiversity awareness.

## 5. Conclusion

Picture books hold potential to raise awareness about animals, which is important considering the widening gap between people and nature. However, the image of animals that is currently conveyed to readers is not very diverse and rather unspecified. Our sample of Dutch award-winning picture books was highly skewed and animals were often visually and textually simplified. Mammals predominated, mainly in essential roles to the storyline, and were specified and anthropomorphized frequently, while animals such as birds, insects, and fish often served to illustrate the environment and were portrayed rather generically. Well-known exotic and domestic species further outnumbered native species. The current representation of animals is likely to both reflect and further skew current perceptions of animals in Western society, and offers children few opportunities to connect with local fauna.

Although artistic freedom of picture book makers is important, we believe that the educational potential of picture books could be tapped into by inspiring illustrators and authors to include a larger diversity of animals in their stories and artwork. Biodiversity professionals could show picture book makers opportunities to diversify. For instance, native species can be easily incorporated in stories set in urbanized environments, which would help dismantle human-nature binaries by making urban children aware that they share the places where they live with wildlife. Even among invertebrates, there are many suitable candidates to portray, as a few books in our sample with striking invertebrate characters (e.g. octopus, stag beetle, peacock butterfly) showed. Moreover, parents and teachers should be encouraged and aided in selecting books that are likely to expand children’s perceptions and that may spark discussion about animal diversity. Ultimately, a diverse and specified portrayal of animals could help foster lasting connections between younger generations and the large variety of animals found on our planet.

## Supplemental Material

sj-docx-1-pus-10.1177_09636625221089811 – Supplemental material for Animal biodiversity and specificity in children’s picture books Supplemental material, sj-docx-1-pus-10.1177_09636625221089811 for Animal biodiversity and specificity in children’s picture books by Michiel Jan Dirk Hooykaas, Marloes Gertrudis Holierhoek, Joris Sebastiaan Westerveld, Menno Schilthuizen and Ionica Smeets in Public Understanding of Science

sj-xlsx-2-pus-10.1177_09636625221089811 – Supplemental material for Animal biodiversity and specificity in children’s picture books Supplemental material, sj-xlsx-2-pus-10.1177_09636625221089811 for Animal biodiversity and specificity in children’s picture books by Michiel Jan Dirk Hooykaas, Marloes Gertrudis Holierhoek, Joris Sebastiaan Westerveld, Menno Schilthuizen and Ionica Smeets in Public Understanding of Science
