# Supplementary material for: Animal biodiversity and specificity in children’s picture books
Source: Public Underst Sci. 2022 May 9;31(5):671–88. doi: 10.1177/09636625221089811 (PMC9131409; doi:10.1177/09636625221089811)
Supplement: sj-docx-1-pus-10.1177_09636625221089811 – Supplemental material for Animal biodiversity and specificity in children’s picture books [file sj-docx-1-pus-10.1177_09636625221089811.docx]

**Supplemental Materials**

Animal Biodiversity and Specificity in Children’s Picture Books

Michiel Jan Dirk Hooykaas^1^, Marloes Gertrudis Holierhoek^1^, Joris Sebastiaan Westerveld^1^, Menno Schilthuizen^1,2^, Ionica Smeets^1^
^1^ Science Communication and Society, Leiden University, 2333 BE Leiden, The Netherlands; m.j.d.hooykaas@biology.leidenuniv.nl (M.J.D.H.); i.smeets@biology.leidenuniv.nl (I.S.)

^2^ Naturalis Biodiversity Center, 2300 RA Leiden, The Netherlands; menno.schilthuizen@naturalis.nl (M.S.)

Correspondence: [m.j.d.hooykaas@biology.leidenuniv.nl](mailto:m.j.d.hooykaas@biology.leidenuniv.nl).

Contents

[Appendix A 2](#_Toc96429402)

[Included Books 2](#_Toc96429403)

[Excluded Books 18](#_Toc96429404)

[Included Awards 20](#_Toc96429405)

[Appendix B 21](#_Toc96429406)

[Codebook 21](#_Toc96429407)

[Appendix C 25](#_Toc96429408)

[Domestic animal species 25](#_Toc96429409)

[Appendix E 26](#_Toc96429410)

[Top 20 most featured animal orders portrayed in children’s picture books (frequency counts for main, supporting, and minor characters, and total). 26](#_Toc96429411)

[Top 20 most featured animal species in children’s picture books (frequency counts for main, supporting, and minor characters, and total). 27](#_Toc96429412)

[Appendix F 28](#_Toc96429413)

[Specificity of text references per class. 28](#_Toc96429414)

[Appendix G: 29](#_Toc96429415)

[Prevalence of different types of anthropomorphism in main, supporting, and minor characters. 29](#_Toc96429416)

[Prevalence of different types of anthropomorphism per class. 29](#_Toc96429417)

# Appendix A

## Included Books

| **book_no** | **title_new** | **title_old** | **year** | **author_name** | **illustrator_name** | **publish_new** | **publish_old** | **publisher_origin** | **prize** |
| --- | --- | --- | --- | --- | --- | --- | --- | --- | --- |
| Each sampled book is given a distinct number. | Title of the book in which the animal is featured. | Original title of the book in which the animal is featured. | Year of publication of the book in which the animal is featured. | Name of the author of the book. | Name of the illustrator of the book. | Name of the publisher of the book published in Dutch. | Name of the original publisher of the book. | Origin of the original publisher of the book in which the animal is featured. | Prize(s) awarded to the picture book |
| e.g. "001", "002", "003"… | e.g. "Panda wil een vriendje" | e.g. "The Only Lonely Panda" | Year (e.g. "2018") | Name | Name | Name | Name | 0=Dutch 1=European 2=Non-European | 1=Picture Book of the Year 2=Flag & Pennants 3=Golden Slate Pencil  4=Silver Slate Pencil 5=Golden Paintbrush 6=Silver Paintbrush 7=Gulden Palet 8=Silver Palet 9=Woutertje Pieterse Prize 10=Children’s Bookshop Prize  If a book received two prices (e.g. Gouden Griffel and Gulden Palet) insert 3,7. |
| 001 | Moppereend | Grumpy Duck | 2018 | Joyce Dunbar | Petr Horácek | Lemniscaat | Walker Books UK | 1 | 1 |
| 002 | Spelen tot het donker wordt | Spelen tot het donker wordt | 2018 | Marit Törnqvist, Hans Hagen, Monique Hagen | Hans Hagen, Monique Hagen | Querido | Querido | 0 | 1 |
| 003 | De hapgrage krokodil | The carnivorous crocodile | 2018 | Jonnie Wild | Brita Granström | de Vries-Brouwers | Otter-Barry Books | 1 | 1 |
| 004 | De ridder zegt nee | The Knight Who Said No! | 2018 | Lucy Rowland | Kate Hindley | Gottmer | Nosy Crow | 1 | 1 |
| 005 | Een schildpad was zijn schildje kwijt | Een schildpad was zijn schildje kwijt | 2018 | Marjet Huiberts | Carmen Saldaña. | Gottmer | Gottmer | 0 | 1 |
| 007 | Zó moe en toch klaarwakker | So Müde und Hellwach | 2019 | Susanne Strasser | Susanne Strasser | Hoogland & Van Klaveren | Peter Hammer Verlag | 1 | 1 |
| 008 | De boer en de dierenarts | De boer en de dierenarts | 2018 | Pim Lammers | Milja Praagman | De Eenhoorn | De Eenhoorn | 1 | 1 |
| 009 | De grote vijf | De grote vijf | 2018 | Bella Makatini | Judi Abbot | Clavis | Clavis | 0 | 1 |
| 011 | Een huis voor Harry | Een huis voor Harry | 2017 | Leo Timmers | Leo Timmers | Querido | Querido | 0 | 1 |
| 012 | Als ik een dinosaurus had | If I had a dinosaur | 2017 | Gabby Dawnay | Alex Barrow | Querido | Thames & Hudson | 1 | 1 |
| 013 | De kale boom | De kale boom | 2017 | Tamara Bos | Barbara de Wolf | De Vier Windstreken | De Vier Windstreken | 0 | 1 |
| 014 | Dino's bestaan niet | Dino's bestaan niet | 2017 | Mark Janssen | Mark Janssen | Lemniscaat | Lemniscaat | 0 | 1.2 |
| 015 | Gulzige geit | The greedy goat | 2017 | Petr Horácek | Petr Horácek | Lemniscaat | Walker Books UK | 1 | 1 |
| 016 | Heb jij misschien Olifant gezien? | Have You Seen Elephant? | 2017 | David Barrow | David Barrow | Gottmer | Gecko Press | 2 | 1.6 |
| 017 | Ossip en de onverwachte reis | Ossip en de onverwachte reis | 2017 | Annemarie van Haeringen | Annemarie van Haeringen | Leopold | Leopold | 0 | 1 |
| 018 | Panda wil een vriendje | The Only Lonely Panda | 2017 | Jonny Lambert | Jonny Lambert | Veltman | Little Tiger Press | 1 | 1 |
| 019 | Plasman | Plasman | 2017 | Jaap Robben | Benjamin Leroy | Gottmer | Gottmer | 0 | 1 |
| 020 | Waar is Beer? | Where's Bear? | 2017 | Emily Gravett | Emily Gravett | Gottmer | Macmillan Children's Books | 1 | 1 |
| 021 | Ssst! De tijger slaapt | Don't wake up the tiger | 2016 | Britta Teckentrup | Britta Teckentrup | Gottmer | Nosy Crow | 1 | 1 |
| 022 | Gewoon zoals je bent | Little Why | 2016 | Jonny Lambert | Jonny Lambert | Veltman | Tiger Tales | 2 | 1 |
| 023 | Slaap maar fijn, bouwterrein | Goodnight, goodnight, construction site | 2016 | Sherri Duskey Rinker | Tom Lichtenfeld | Moon | Chronicle Books | 2 | 1 |
| 024 | Antonia | Antonia | 2016 | Anke de Vries | Piet Grobler | Lemniscaat | Lemniscaat | 0 | 1 |
| 025 | De kusjeskrokodil | De kusjeskrokodil en andere lieve nachtbeesten | 2016 | Jozua Douglas | Loes Riphagen | De Fontein | Clavis | 0 | 1 |
| 026 | Alberts Boom | Albert's Tree | 2016 | Jenni Desmond | Jenni Desmond | Lemniscaat | Walker Books UK UK | 1 | 1 |
| 027 | Ik ben bij de dinosaurussen geweest | Ik ben bij de dinosaurussen geweest | 2016 | Edward van de Vendel | Floor de Goede | Querido | Querido | 0 | 1.2 |
| 028 | Mijn potje | Mijn potje | 2016 | Anita Bijsterbosch | Anita Bijsterbosch | Clavis | Clavis | 0 | 1 |
| 029 | Mmm… een taart! | Mmm… een taart! | 2016 | Susanne Strasser | Susanne Strasser | Hoogland & Van Klaveren | Hoogland & Van Klaveren | 0 | 1 |
| 030 | Nog even mijn haartjes wassen | Badetag fur Hasenkind | 2016 | Jörg Mühle | Jörg Mühle | Gottmer | Moritz Verlag | 1 | 1 |
| 031 | De kleine walvis | The storm whale | 2015 | Benji Davies | Benji Davies | Luitingh-Sijthoff | Simon & Schuster Children's UK | 1 | 1 |
| 032 | Berre is bijna jarig | Berre is bijna jarig | 2015 | Annemie Berebrouckx | Annemie Berebrouckx | De Eenhoorn | De Eenhoorn | 1 | 1 |
| 033 | Buurman Brombeer | Those pesky rabbits | 2015 | Ciara Flood | Ciara Flood | De Fontein | Templar Publishing | 1 | 1 |
| 034 | Hé, wie zit er op de wc? | Hé, wie zit er op de wc? | 2015 | Harmen van Straaten | Harmen van Straaten | Leopold | Leopold | 0 | 1 |
| 036 | Klein Geitje kan alles | Klein Geitje kan alles | 2015 | Yvonne Jagtenberg | Yvonne Jagtenberg | Leopold | Leopold | 0 | 1 |
| 037 | Kleine blauwe truck | Little blue truck | 2015 | Alice Schertle | Jill Mcelmurry | Gottmer | Houghton Mifflin Harcourt | 2 | 1 |
| 038 | Kom uit die kraan! | Kom uit die kraan! | 2015 | Tjibbe Veldkamp | Alice Hoogstad | Lemniscaat | Lemniscaat | 0 | 1.4 |
| 039 | Lente, zomer, herfst, winter | Tree | 2015 | Pat Hegarty | Britta Teckentrup | Veltman | Little Tiger Press | 1 | 1 |
| 041 | De krijtjes staken | The day the crayons quit | 2014 | Oliver Jeffers | Drew Daywalt | De Fontein | Philomel Books | 2 | 1.4 |
| 042 | De krokodil die niet van water hield | The Crocodile Who Didn't Like Water | 2014 | Gemma Merino | Gemma Merino | Lemniscaat | Macmillan Children's Books | 1 | 1 |
| 043 | De leeuw en het vogeltje | Le lion et l'oiseau | 2014 | Marianne Dubuc | Marianne Dubuc | Querido | La Pastèque | 2 | 1 |
| 044 | Feestmaal voor de koning | Feestmaal voor de koning | 2014 | Marlies Verhelst | Linde Faas | Lemniscaat | Lemniscaat | 0 | 1 |
| 045 | Gewonnen | Gewonnen | 2014 | Ruth Wielockx | Ruth Wielockx | Clavis | Clavis | 0 | 1 |
| 046 | Gonnie & vriendjes in ganzenpas | Gonnie and friends […] | 2014 | Olivier Dunrea | Olivier Dunrea | Gottmer | Houghton Mifflin Harcourt | 2 | 1 |
| 047 | Het ei van Egel | L'oeuf d'Hérisson | 2014 | Nozomi Takahashi | Nozomi Takahashi | De Eenhoorn | Lirabelle | 1 | 1 |
| 048 | Sneeuwwitje breit een monster | Sneeuwwitje breit een monster | 2014 | Annemarie van Haeringen | Annemarie van Haeringen | Leopold | Leopold | 0 | 1 |
| 049 | Vergeet mij nietje | Vergeet mij nietje | 2014 | Milja Praagman | Milja Praagman | De Eenhoorn | De Eenhoorn | 1 | 1 |
| 050 | We hebben er een geitje bij! | We hebben er een geitje bij! | 2014 | Marjet Huiberts | Iris Deppe | Gottmer | Gottmer | 0 | 1 |
| 051 | Boer Boris gaat naar zee | Boer Boris gaat naar zee | 2014 | Ted van Lieshout | Philip Hopman | Gottmer | Gottmer | 0 | 1 |
| 052 | Als iedereen slaapt |  | 2013 | Komako Sakai | Komako Sakai | De Eenhoorn | 99 | 2 | 1.2 |
| 053 | Het boekenliefje | The Snatchabook | 2013 | Helen Docherty & Thomas Docherty | Helen Docherty | Clavis | Green Books | 1 | 1 |
| 054 | Ik wil ook! | Ik wil ook! | 2013 | Bibi Dumon Tak & Annemarie van Haeringen | Annemarie van Haeringen | Querido | Querido | 0 | 1 |
| 055 | Klop klop klop | Peck, peck, peck | 2013 | Lucy Cousins | Lucy Cousins | Leopold | Walker Books UK | 1 | 1 |
| 056 | Koning Koen en de draak | King Jack and the Dragon Book | 2013 | Peter Bently | Helen Oxenbury | De Vier Windstreken | Penguin Random House Children's UK | 1 | 1 |
| 057 | Pas op! Dit boek bijt! | Open Very Carefully | 2013 | Nick Bromley | Nicola O’Byrne | Gottmer | Nosy Crow | 1 | 1 |
| 058 | Piepkleine muis | The Tiny Mouse | 2013 | Janis Ian | Ingrid Schubert, Dieter Schubert | Lemniscaat | Lemniscaat USA | 2 | 1 |
| 059 | Piep wil oversteken | Piep wil oversteken | 2013 | Fleur van der Weel | Fleur van der Weel | Singel | Singel | 0 | 1 |
| 060 | Snel naar huis, kleine muis | Lauf nach Haus, Kleine Maus | 2013 | Britta Teckentrup | Britta Teckentrup | Gottmer | Jacoby & Stuart | 1 | 1 |
| 061 | Krrrr… okodil! | Solomon crocodile | 2012 | Catherine Rayner | Catherine Rayner | Vries-Brouwers | Macmillan Children's Books | 1 | 1 |
| 062 | Emilie! Eten! | Lunchtime | 2012 | Rebecca Cobb | Rebecca Cobb | Vries-Brouwers | Macmillan Children's Books | 1 | 1 |
| 063 | Het boek van Max | Little Nelly's big book | 2012 | Pippa Goodhart | Andy Rowland | De Fontein | Bloomsbury USA | 2 | 1 |
| 064 | Boer Boris | Boer Boris | 2012 | Ted van Lieshout | Philip Hopman | Gottmer | Gottmer | 0 | 1 |
| 065 | Pip en Posy en de grote ballon | The big balloon | 2012 | Axel Scheffler | Axel Scheffler | Gottmer | Nosy Crow | 1 | 1 |
| 066 | Knuffelbunny | Knuffle Bunny | 2012 | Mo Willems | Mo Willems | Gottmer | Walker Books UK | 1 | 1 |
| 068 | En hoe komt toch een olifant aan al die kilo’s grijs? | En hoe komt toch een olifant aan al die kilo’s grijs? | 2012 | Elle van Lieshout, Erik van Os | Alice Hoogstad | Lemniscaat | Lemniscaat | 0 | 1 |
| 069 | Balotje bij de dokter | Balotje bij de dokter | 2012 | Yvonne Jagtenberg | Yvonne Jagtenberg | Leopold | Leopold | 0 | 1 |
| 070 | Maak je niet dik! | Don't worry Douglas! | 2012 | David Melling | David Melling | Van Goor | Hodder Children's Books | 1 | 1 |
| 071 | Nog 100 nachtjes slapen | Nog 100 nachtjes slapen | 2011 | Milja Praagman | Milja Praagman | Leopold | Leopold | 0 | 1 |
| 072 | Ik wil een knuffel | Hugless Douglas | 2011 | David Melling | David Melling | Van Goor | Hodder Children's Books | 1 | 1 |
| 073 | Gek hondje | Silly doggy | 2011 | Adam Stower | Adam Stower | Vries-Brouwers | Templar Publishing | 1 | 1 |
| 074 | Superbeesje is al onderweg | Superbeesje is al onderweg | 2011 | Guido van Genechten | Guido van Genechten | Clavis | Clavis | 0 | 1 |
| 075 | Neushoorns eten geen pannenkoeken | Rhinos don't eat pancakes | 2011 | Anna Kemp | Sara Ogilvie | Lemniscaat | Simon & Schuster | 1 | 1 |
| 076 | Er was eens een vosje… | Er was eens een vosje… | 2011 | Gitte Spee | Gitte Spee | Moon | Moon | 0 | 1 |
| 077 | Agent en Boef en de Boefagent | Agent en Boef en de Boefagent | 2011 | Tjibbe Veldkamp | Kees de Boer | Lannoo | Lannoo | 0 | 1 |
| 078 | Waarom lig jij in mijn bedje | Waarom lig jij in mijn bedje | 2011 | Joke van Leeuwen | Joke van Leeuwen | Singel | Singel | 0 | 1.2 |
| 079 | Pieter de papegaaiduiker | Puffin Peter | 2011 | Petr Horácek | Petr Horácek | De Vier Windstreken | Walker Books UK | 1 | 1 |
| 081 | Mama kwijt | Little Owl Lost | 2010 | Chris Haughton | Chris Haughton | Gottmer | Walker Books UK | 1 | 1 |
| 082 | Ik ben de beste | I'm the Best | 2010 | Lucy Cousins | Lucy Cousins | Leopold | Walker Books UK | 1 | 1 |
| 083 | Wat het lieveheersbeestje hoorde | What the ladybird heard | 2010 | Julia Donaldson | Lydia Monks | Gottmer | Macmillan Children's Books | 1 | 1 |
| 084 | Zaza speelt doktertje | Zaza speelt doktertje | 2010 | Mylo Freeman | Mylo Freeman | Clavis | Clavis | 0 | 1 |
| 086 | Ikke niet | Not Me! | 2010 | Nicolas Killen | Nicolas Killen | Luister | Egmont Books UK | 1 | 1 |
| 088 | Vliegensvlugge vlieg | Tiny Little Fly | 2010 | Michael Rosen | Kevin Waldron | Lemniscaat | Walker Books UK | 1 | 1 |
| 089 | Bramenjam | Bramenjam | 2010 | Gerda Baardman | Natascha Stenvert | Moon | Moon | 0 | 1 |
| 090 | Meneer René | Meneer René | 2010 | Leo Timmers | Leo Timmers | Querido | Querido | 0 | 1 |
| 091 | Fiet wil rennen | Fiet wil rennen | 2009 | Bibi Dumon Tak | Noëlle Smit | Singel | Singel | 0 | 1.4 |
| 092 | Boe! roept Klein Konijn | The Little Rabbit Who Liked to Say Moo | 2009 | Jonathan Allen | Jonathan Allen | van Goor | Boxer Books | 2 | 1 |
| 093 | Waar is mijn sok? | Waar is mijn sok? | 2009 | Marijke ten Cate | Marijke ten Cate | Lemniscaat | Lemniscaat | 0 | 1 |
| 094 | Bout en Moertje | Bout en Moertje | 2009 | Nicole de Cock | Nicole de Cock | Gottmer | Gottmer | 0 | 1 |
| 095 | Naar het strand | To the Beach | 2009 | Thomas Docherty | Thomas Docherty | van Goor | Templar Publishing | 1 | 1 |
| 097 | Balotje en het tasje van oma | Balotje en het tasje van oma | 2009 | Yvonne Jagtenberg | Yvonne Jagtenberg | Leopold | Leopold | 0 | 1 |
| 098 | O, o Octopus | O, o Octopus | 2009 | Elle van Lieshout, Erik van Os | Mies van Hout | Lemniscaat | Lemniscaat | 0 | 1 |
| 099 | Ik moet zó nodig! | Ik moet zó nodig! | 2009 | Fiona Rempt | Noëlle Smit | van Goor | van Goor | 0 | 1 |
| 100 | De coole cowboy | De coole cowboy | 2009 | Tjibbe Veldkamp & Wouter Tulp | Wouter Tulp | van Goor | van Goor | 0 | 1 |
| 101 | De Wiebelbillenboogie | De Wiebelbillenboogie | 2008 | Guido Van Genechten | Guido Van Genechten | Clavis | Clavis | 0 | 1 |
| 102 | De koe die een ei legde | The Cow That Laid an Egg | 2008 | Andy Cutbill | Russel Ayto | Lemniscaat | Harper Collins | 1 | 1 |
| 103 | Giraf heeft het koud | Giraf heeft het koud | 2008 | Judith Koppens | Judith Koppens | Clavis | Clavis | 0 | 1 |
| 104 | Ik wil een hond | The Best Pet of All | 2008 | David LaRochelle | Hanako Wakiyama | Lemniscaat | Dutton Books For Young Readers | 2 | 1 |
| 105 | Niels wil nog niet slapen | Ab ins Bett, Nils! | 2008 | Marcus Pfister | Marcus Pfister | De Vier Windstreken | NordSüd | 1 | 1 |
| 106 | Ik voel een voet! | Ik voel een voet! | 2008 | Maranke Rinck | Martijn van der Linden | Lemniscaat | Lemniscaat | 0 | 1 |
| 107 | Jarig | Jarig | 2010 | Liesbet Slegers | Liesbet Slegers | Clavis | Clavis | 0 | 1 |
| 108 | Agent en boef | Agent en boef | 2008 | Tjibbe Veldkamp | Kees de Boer | Lannoo | Lannoo | 0 | 1 |
| 109 | Raf | Raf | 2008 | Anke de Vries | Charlotte Dematons | Lemniscaat | Lemniscaat | 0 | 1 |
| 110 | Tandenpoetsen! | Tandenpoetsen! | 2008 | Helga Warmels | Barbara de Wolf | Mercis | Mercis | 0 | 1 |
| 111 | Mijn wonderlijke oom | Mijn wonderlijke oom | 2019 | Yvonne Jagtenberg | Yvonne Jagtenberg | Rubinstein | Rubinstein | 0 | 5.6 |
| 112 | De wolf, de eend en de muis | De wolf, de eend en de muis | 2019 | Mac Barnett | Jon Klassen | Gottmer | Candlewick Press | 2 | 2.4 |
| 113 | Die eland is van mij | This Moose Belongs to Me | 2018 | Oliver Jeffers | Oliver Jeffers | Hoogland & Van Klaveren | Harper Collins | 1 | 4 |
| 114 | Droomopa | Droomopa | 2019 | Dolf Verroen | Charlotte Dematons | Leopold | Leopold | 0 | 4 |
| 115 | Vosje | Vosje | 2018 | Edward van de Vendel | Marije Tolman | Querido | Querido | 0 | 4.6 |
| 116 | Van twee ridders | Van twee ridders | 2019 | Imme Dros | Harry Geelen | Querido | Querido | 0 | 6 |
| 117 | De pittige pruim die een pop werd | Panáček, pecka, švestka, poleno a zase panáček | 2018 | Vojtěch Mašek | Chrudoš Valoušek | Boycott Books | Baobab | 1 | 6 |
| 118 | Johannes de parkiet | Johannes de parkiet | 2018 | Mark Haayema | Medy Oberendorff | Rubinstein | Rubinstein | 0 | 2 |
| 119 | Het meisje en haar zeven paarden | 99 | 2018 | Hadi Mohammadi | Nooshin Safakhoo | Querido | 99 | 2 | 2 |
| 120 | Woesssj! | Sweep | 2018 | Louise Greig | Julia Sarda | C. de Vries-Brouwers | Egmont Books UK | 1 | 2 |
| 122 | De schelmenstreken van Reinaert de Vos | De schelmenstreken van Reinaert de Vos | 2018 | Koos Meinderts | Carl Cneut, Annemarie van Haeringen, Charlotte Dematons, Alice Hoogstad, Annette Fienieg, Piet Grobler, Mies van Hout, Martijn van der Linden, Sanne te Loo, Daan Remmert de Vries, Ingrid Schubert, Dieter Schubert, Hanneke Siemensma, Noelle Smit, Harmen van Straaten, The Tjong-Khing, Marije Tolman, Ludwig Volbeda, Fleur van der Weel, Sylvia Weve | Hoogland & van Klaveren | Hoogland & van Klaveren | 0 | 2 |
| 123 | Liefde is niet voor lafaards | En liten bok om kärlek | 2018 | Ulf Stark | Ida Björns | Querido | Lilla Piratförlaget | 1 | 2 |
| 124 | Als Verdriet op bezoek komt | Als Verdriet op bezoek komt | 2018 | Eva Eland | Eva Eland | Leopold | Leopold | 0 | 2 |
| 125 | Een indiaan als jij en ik | Een indiaan als jij en ik | 2018 | Erna Sassen | Martijn van der Linden | Leopold | Leopold | 0 | 2 |
| 129 | Het lammetje dat een varken was | Het lammetje dat een varken was | 2017 | Pim Lammers | Milja Praagman | De Eenhoorn | De Eenhoorn | 1 | 4 |
| 130 | Handje? | Handje? | 2017 | Tjibbe Veldkamp | Wouter Tulp | De Fontein | De Fontein | 0 | 4 |
| 131 | Toen ik | Toen ik | 2017 | Joke van Leeuwen | Joke van Leeuwen | Querido | Querido | 0 | 4 |
| 132 | Het gelukkige eiland | Het gelukkige eiland | 2017 | Marit Törnqvist | Marit Törnqvist | Querido | Querido | 0 | 4 |
| 134 | Dit is voor jou | Dit is voor jou | 2017 | Sanne te Loo | Sanne te Loo | Lemniscaat | Lemniscaat | 0 | 6 |
| 135 | Konijnentango | Konijnentango | 2017 | Daan Remmerts de Vries | Ingrid Schubert, Dieter Schubert | Hoogland & Van Klaveren | Hoogland & Van Klaveren | 0 | 6 |
| 136 | Verliefd | Verliefd | 2017 | Stefan Boonen | Jan van Lierde | Van Halewyck | Van Halewyck | 1 | 2 |
| 137 | Alle dieren drijven | Noah und die große Flut | 2017 | Gideon Samson | Annemarie van Haeringen | Leopold (ook tegelijk uitgegeven via Duitse Gerstenberg Verlag) | Leopold (ook tegelijk uitgegeven via Duitse Gerstenberg Verlag) | 0 | 2 |
| 138 | Kersenhemel | Kersenhemel | 2017 | Jef Aerts | Sanne te Loo | Querido | Querido | 0 | 2 |
| 140 | De cycloop | De cycloop | 2017 | Daan Remmerts de Vries | Floor Rieder | Gottmer | Gottmer | 0 | 2 |
| 141 | Zondag, maandag, sterrendag | Zondag, maandag, sterrendag | 2017 | Anna Woltz | Annet Schaap | Querido | Querido | 0 | 2 |
| 142 | Driehoek | Triangle | 2017 | Mac Barnett | Jon Klassen | Gottmer | Candlewick Press | 2 | 2 |
| 144 | Kleine nachtverhalen | Petites histoires de nuits (onduidelijk of deze of Nederlandse titel eerder was) | 2017 | Kitty Crowther | Kitty Crowther | De Eenhoorn | Lilla piratförlaget | 1 | 2 |
| 145 | Voor Papa | Voor Papa | 2017 | Daan Remmerts de Vries | Marije Tolman | Hoogland & Van Klaveren | Hoogland & Van Klaveren | 0 | 2 |
| 146 | De Vos en de Ster | Fox and the star | 2017 | Coralie Bickford-Smith | Coralie Bickford-Smith | Christofoor | Penguin Random House Children's UK | 1 | 2 |
| 147 | Anne, het paard en de rivier | Anne, het paard en de rivier | 2017 | Wouter Klootwijk | Enzo Pérèz-Labourdette | Leopold | Leopold | 0 | 2 |
| 148 | Tangramkat | Tangramkat | 2016 | Martijn van der Linden | Martijn van der Linden | Lemniscaat | Lemniscaat | 0 | 4.5 |
| 149 | Circusnacht | Circusnacht | 2016 | Mattias De Leeuw | Mattias De Leeuw | Lannoo | Lannoo | 1 | 7 |
| 150 | Siens hemel | Siens hemel | 2016 | Bibi Dumon Tak | Annemarie van Haeringen | Querido | Querido | 0 | 4 |
| 152 | Omdat ik je zo graag zie | Omdat ik je zo graag zie | 2016 | Milja Praagman | Milja Praagman | De Eenhoorn | De Eenhoorn | 1 | 6 |
| 153 | Kek iz tak? | Du iz tak | 2016 | Carson Ellis | Carson Ellis | Leopold | Candlewick Press | 2 | 8 |
| 154 | Een vriendje voor altijd | Imaginary Fred | 2016 | Eoin Colfer | Oliver Jeffers | De Fontein | Harper Collins | 1 | 2 |
| 155 | De tuinman van de nacht | The Night Gardener | 2016 | The Fan Brothers | The Fan Brothers | Leopold | Simon & Schuster | 2 | 2 |
| 156 | Jan Toorop - Het lied van de tijd | Jan Toorop - Het lied van de tijd | 2016 | Kitty Crowther | Kitty Crowther | Leopold | Leopold | 0 | 2 |
| 157 | Wauw Pauw | Wauw Pauw | 2016 | Yoko Heiligers | Mariken Jongman | Marmer | Marmer | 0 | 2 |
| 158 | Lettersoep | Lettersoep | 2015 | Harriët van Reek | Harriët van Reek | Querido | Querido | 0 | 5 |
| 159 | Het gat | Hullet | 2015 | Øyvind Torseter | Øyvind Torseter | de Harmonie | Cappelen Damm | 1 | 7 |
| 160 | Tijs en de eenhoorn | Tijs en de eenhoorn | 2015 | Imme Dros | Harrie Geelen | Querido | Querido | 0 | 4 |
| 162 | De tuin van de walvis | De tuin van de walvis | 2015 | Toon Telligen | Annemarie van Haeringen | Querido | Querido | 0 | 4 |
| 163 | Hondje, de enige echte | Hondje, de enige echte | 2015 | Yvonne Jagtenberg | Yvonne Jagtenberg | Rubinstein | Rubinstein | 0 | 6 |
| 164 | Mijn opa is een boom | Mijn opa is een boom | 2015 | Kim Cabreels | Ingrid Godon | Lannoo | Lannoo | 1 | 8 |
| 165 | Bas & Daan graven een gat | Sam & Dave dig a hole | 2015 | Mac Barnett | Jon Klassen | Hoogland & Van Klaveren | Candlewick Press | 2 | 2 |
| 166 | Wat zou jij doen? | Wat zou jij doen? | 2015 | Guido Van Genechten | Guido Van Genechten | Clavis | Clavis | 0 | 2 |
| 167 | Bens boot | Bens boot | 2016 | Pieter Koolwijk | Linde Faas | Lemniscaat | Lemniscaat | 0 | 2 |
| 168 | Lotte & Roos. Samen ben je niet alleen | Lotte & Roos. Samen ben je niet alleen | 2015 | Marieke Smithuis | Annet Schaap | Querido | Querido | 0 | 2 |
| 171 | Schobbejacques en de 7 geiten | Schobbejacques en de 7 geiten | 2015 | Merlijne Marell | Merlijne Marell | Loopvis | Loopvis | 0 | 2 |
| 173 | Monsterboek | Monsterboek | 2014 | Alice Hoogstad | Alice Hoogstad | Lemniscaat | Lemniscaat | 0 | 5.6 |
| 175 | Soms laat ik je even achter | Soms laat ik je even achter | 2014 | Daan Remmerts de Vries | Daan Remmerts de Vries | Querido | Querido | 0 | 4 |
| 176 | Bruno wordt een superheld | Brune | 2014 | Håkon Øvreås | Øyvind Torseter | Querido | Gyldendal | 1 | 4 |
| 177 | Een afspraakje in het bos | Een afspraakje in het bos | 2014 | Sylvia Vanden Heede | Benjamin Leroy | Lannoo | Lannoo | 1 | 4 |
| 179 | Het donker | The dark | 2014 | Lemony Snicket | Jon Klassen | Gottmer | Hachette Children's Group - Little, Brown Books for Young Readers | 2 | 8 |
| 180 | Kietel nooit een krokodil | Kietel nooit een krokodil | 2014 | Bette Westera | Thé Tjong-Khing | Gottmer | Gottmer | 0 | 2 |
| 181 | Hoe je een wollige mammoet moet wassen - in tien lessen | How to wash a woolly mammoth? | 2013 | Michelle Robinson | Kate Hindley | Querido | Simon & Schuster | 1 | 2 |
| 182 | De wondertuin | De wondertuin | 2014 | Gerda Dendooven | Gerda Dendooven | Querido | Querido | 0 | 2 |
| 185 | Het hondje dat Nino niet had | Het hondje dat Nino niet had | 2013 | Edward van de Vendel | Anton van Hertbrugge | De Eenhoorn | De Eenhoorn | 1 | 7 |
| 186 | Held op sokken | Held op sokken | 2013 | Bette Westera | Thé Tjong-Khing | Gottmer | Gottmer | 0 | 4 |
| 187 | Wij samen op stap | All Through My Town | 2013 | Jean Reidy | Leo Timmers | Querido | Bloomsbury USA | 2 | 4 |
| 188 | Garmanns straat | Garmanns gate | 2013 | Stian Hole | Stian Hole | Hoogland & Van Klaveren | Cappelen Damm | 1 | 4 |
| 189 | Groter dan een droom | Groter dan een droom | 2013 | Jef Aerts | Marit Törnqvist | Querido | Querido | 0 | 4 |
| 190 | Coco of het kleine zwarte jurkje | Coco of het kleine zwarte jurkje | 2013 | Annemarie van Haeringen | Annemarie van Haeringen | Leopold | Leopold | 0 | 6 |
| 191 | Deze hoed is niet van mij | This is not my hat | 2013 | Jon Klassen | Jon Klassen | Gottmer | Walker Books UK | 1 | 8 |
| 192 | Boer Boris in de sneeuw | Boer Boris in de sneeuw | 2013 | Ted van Lieshout | Philip Hopman | Gottmer | Gottmer | 0 | 2 |
| 193 | Prinses Pernilla en de reddende ridders | Prinses Pernilla en de reddende ridders | 2013 | Mathilde Stein | Dorine de Vos | Lemniscaat | Lemniscaat | 0 | 2 |
| 194 | Jonas en de visjes van Kees Poon | Jonas en de visjes van Kees Poon | 2012 | Harm de Jonge | Martijn van der Linden | Hoogland & Van Klaveren | Hoogland & Van Klaveren | 0 | 2 |
| 195 | Sammie en opa | Sammie en opa | 2013 | Enne Koens | Kees de Boer | Moon | Moon | 0 | 2 |
| 196 | Vlieg! | Vlieg! | 2013 | Marco Kunst | Philip Hopman | Lemniscaat | Lemniscaat | 0 | 2 |
| 199 | Zondag | Søndag | 2012 | Kim Fupz Aakeson | Eva Eriksson | Querido | Gyldendal | 1 | 4 |
| 200 | Zoveel als de wereld hou ik van jou | Zoveel als de wereld hou ik van jou | 2012 | Imme Dros | Harrie Geelen | Querido | Querido | 0 | 4 |
| 201 | Springdag | Springdag | 2012 | Anne Provoost | An Candaele | De Eenhoorn | De Eenhoorn | 1 | 4 |
| 202 | Wie klopt daar? | Wie klopt daar? | 2012 | Bart Moeyaert | Gerda Dendooven | De Eenhoorn | De Eenhoorn | 1 | 4 |
| 204 | Takkenkind | Takkenkind | 2012 | Gerda Dendooven | Gerda Dendooven | Querido | Querido | 0 | 8 |
| 205 | Buurman leest een boek | Buurman leest een boek | 2012 | Koen van Biesen | Warre Borgmans | De Eenhoorn | De Eenhoorn | 1 | 2 |
| 206 | Waar was Hans? | Waar was Hans? | 2012 | Ienne Biemans | Ceseli Josephus Jitta | Gottmer | Gottmer | 0 | 2 |
| 207 | Het hanengevecht | Het hanengevecht | 2012 | Hans & Monique Hagen | Philip Hopman | Querido | Querido | 0 | 2 |
| 208 | Camping Zeevos | Camping Zeevos | 2012 | Hilde Vandermeerderen | Harmen van Straaten | Davidsfonds | Davidsfonds | 1 | 2 |
| 209 | Meneertje Streepjespyjama in New York |  | 2012 | Michaël Leblond | Frédérique Bertrand | Clavis | Phoenix Yard Books | 1 | 2 |
| 212 | Kleine Man en God | Le petit homme et Dieu | 2011 | Kitty Crowther | Kitty Crowther | De Eenhoorn | Pastel L'école des loisirs | 1 | 7.8 |
| 213 | Keepvogel en Kijkvogel | Keepvogel en Kijkvogel in het spoor van Mondriaan | 2011 | Wouter van Reek | Wouter van Reek | Leopold | Leopold | 0 | 2.4 |
| 214 | O rode papaver | O rode papaver, boem pats knal! | 2011 | Sjoerd Kuyper | Marije Tolman | Lemniscaat | Lemniscaat | 0 | 4 |
| 216 | Toen kwam Sam | Toen kwam Sam | 2011 | Edward van de Vendel | Philip Hopman | Querido | Querido | 0 | 4 |
| 217 | Het Muizenhuis - Sam & Julia | Het Muizenhuis - Sam & Julia | 2011 | Karina Schaapman | Ton Bower | Rubinstein | Rubinstein | 0 | 6 |
| 218 | Iggy en ik | Iggy and me | 2011 | Jenny Valentine | Sandra Klaassen | Moon | Harper Collins | 1 | 2 |
| 219 | Lieve kleine Rolf | Good little wolf | 2011 | Nadia Shireen | Nadia Shireen | C. de Vries-Brouwers | Jonathan Cape | 1 | 2 |
| 220 | Beste Bregje Boentjes | Beste Bregje Boentjes | 2011 | Mathilde Stein | Chuck Groenink | Lemniscaat | Lemniscaat | 0 | 2 |
| 221 | Mees Kees gaat verhuizen | Mees Kees gaat verhuizen | 2011 | Mirjam Oldenhave | Rick de Haas | Ploegsma | Ploegsma | 0 | 2 |
| 222 | Mijn opa en ik en het varken Oma | Mijn opa en ik en het varken Oma | 2011 | Marjolijn Hof | Judith ten Bosch | Querido | Querido | 0 | 2 |
| 223 | Papa hoor je me | Papa hoor je me | 2011 | Tamara Bos | Annemarie van Haeringen | Leopold | Leopold | 0 | 2 |
| 224 | Juffrouw van Zanten en de zeven rovers | Juffrouw van Zanten en de zeven rovers | 2011 | Mathilde Stein | Dorine de Vos | Lemniscaat | Lemniscaat | 0 | 2 |
| 226 | En? | Alors? | 2010 | Kitty Crowther | Kitty Crowther | De Eenhoorn | L'école des loisirs | 1 | 4 |
| 227 | Het boeboek | Het boeboek | 2010 | Imme Dros | Harrie Geelen | Querido | Querido | 0 | 4 |
| 229 | Hoe oma almaar kleiner werd | Hoe oma almaar kleiner werd | 2010 | Michael de Cock | Kristien Aertssen | Querido | Querido | 0 | 4 |
| 230 | Angèle de Verschrikkelijke | Angèle de Verschrikkelijke | 2010 | Tine Mortier | Bert Dombrecht | De Eenhoorn | De Eenhoorn | 1 | 2 |
| 231 | Beu | Beu | 2010 | Kaat Vrancken | Noëlle Smit | Querido | Querido | 0 | 2 |
| 232 | Mees Kees - De sponsorloop | Mees Kees - De sponsorloop | 2010 | Mirjam Oldenhave | Rick de Haas | Ploegsma | Ploegsma | 0 | 2 |
| 233 | Meneer G. | El Señor G. | 2010 | Gustavo Roldán | Gustavo Roldán | Van Goor | A Buen Paso | 1 | 2 |
| 234 | Rosie en Moussa | Rosie en Moussa | 2010 | Michael de Cock | Judith van Istendael | Querido | Querido | 0 | 2 |
| 235 | De staart van meneer kat | De staart van meneer kat | 2010 | Tjalling Houkema | Tjalling Houkema | Gottmer | Gottmer | 0 | 2 |
| 236 | Voordat jij er was | Voordat jij er was | 2009 | Daan Remmerts de Vries | Philip Hopman | Querido | Querido | 0 | 2,3,4 |
| 237 | De boomhut | De boomhut | 2009 | Marije Tolman | Ronald Tolman | Lemniscaat | Lemniscaat | 0 | 5 |
| 239 | Hoe het varken aan zijn krulstaart kwam | Hoe het varken aan zijn krulstaart kwam | 2009 | Gerda Dendooven | Gerda Dendooven | Querido | Querido | 0 | 4 |
| 240 | Grote wolf en kleine wolf | Grand Loup et Petit Loup | 2010 | Nadine Brun-Cosme | Olivier Tallec | Hoogland & Van Klaveren | Flammarion | 1 | 2 |
| 241 | Kleine ezel en de durfal | Kleine ezel en de durfal | 2009 | Rindert Kromhout | Annemarie van Haeringen | Leopold | Leopold | 0 | 2 |
| 242 | Het land van de grote woordfabriek | La grande fabrique de mots | 2009 | Agnès de Lestrade | Valeria Docampo | De Eenhoorn | Alice Editions | 1 | 2 |
| 243 | Al zijn eendjes | Alle seine Entlein | 2009 | Christian Duda | Julia Friese | Gottmer | Bajazzo Verlag | 1 | 2 |
| 244 | Wolf en Hond | Wolf en Hond | 2009 | Sylvia Vanden Heede | Marije Tolman | Lannoo | Lannoo | 1 | 2 |
| 245 | Op zoek naar opa Bleskop | Op zoek naar opa Bleskop | 2008 | Margriet Heymans | Margriet Heymans | Querido | Querido | 0 | 2 |
| 247 | Een wel heel bijzondere kerst | Een wel heel bijzondere kerst | 2010 | Kristien in-'t-Ven | Geert Vervaeke | Lannoo | Lannoo | 1 | 6 |
| 248 | Huisbeestenboel | Huisbeestenboel | 2009 | Loes Riphagen | Loes Riphagen | De Fontein | De Fontein | 0 | 6 |
| 249 | Stella, ster van de zee | Stella, ster van de zee | 2016 | Gerda Dendooven | Gerda Dendooven | Querido | Querido | 0 | 9 |
| 251 | Eiland | Eiland | 2019 | Mark Janssen | Mark Janssen | Lemniscaat | Lemniscaat | 0 | 10 |
| 252 | Bij ons in de straat | Bij ons in de straat | 2012 | Koos Meinderts | Annette Fienieg | Lemniscaat | Lemniscaat | 0 | 10 |
| 254 | Poten omhoog | Poten omhoog | 2010 | Catharina Valckx | Catharina Valckx | Gottmer | Gottmer | 0 | 10 |

## Excluded Books

| **book_no** | **title_new** | **title_old** | **publish_new** | **prize** | **reason for exclusion** |
| --- | --- | --- | --- | --- | --- |
| Each sampled book is given a distinct number. | Title of the book in which the animal is featured. | Original title of the book in which the animal is featured. | Name of the publisher of the book published in Dutch. | Prize(s) awarded to the picture book |  |
| e.g. "001", "002", "003"… | e.g. "Panda wil een vriendje" | e.g. "The Only Lonely Panda" | Name | 1=Picture Book of the Year 2=Flag & Pennants 3=Golden Slate Pencil  4=Silver Slate Pencil 5=Golden Paintbrush 6=Silver Paintbrush 7=Gulden Palet 8=Silver Palet 9=Woutertje Pieterse Prize 10=Children’s Bookshop Prize  If a book received two prices (e.g. Gouden Griffel and Gulden Palet) insert 3,7. |  |
| 006 | Van 1 tot 10 | Mies van Hout | Gottmer | 1 | non-storybook |
| 010 | Otto groot Otto klein: 101 tegenstellingen | Tom Schamp | Lannoo | 1 | non-storybook |
| 035 | Het boek zonder tekeningen | Benjamin Joseph Manaly | Lanno | 1.2 | book without illustrations |
| 040 | Van wie is die hoed? | Joukje Akveld | Gottmer | 1.4 | non-storybook |
| 067 | Gewoon gek! | Ingrid Schuber, Dieter Schubert | Lemniscaat | 1.4 | non-storybook |
| 085 | Daar buiten loopt een schaap | Mies van Hout | Lemniscaat | 1 | omnibus edition |
| 087 | Netjes wachten in de rij | Tomoko Ohmura | Gottmer | 1 | non-storybook |
| 096 | Wie? | Guido van Genechten | Clavis | 1 | non-storybook |
| 121 | De kikkerbilletjes van de koning | Janneke Schotveld | Van Holkema & Warendorf | 2 | omnibus edition |
| 126 | Laat een boodschap achter in het zand | Bibi Dumon Tak | Querido | 2.4 | omnibus (poems) |
| 127 | Zo kreeg Midas ezelsoren | Maria van Donkelaar | Gottmer | 2.4 | omnibus edition |
| 128 | Fabeldieren | Floortje Zwigtman | Lannoo | 2,5,6,9 | non-storybook |
| 133 | Pippeloentje | Annie M.G. Schmidt | Querido | 6 | omnibus (poems) |
| 139 | Bedtijdverhalen voor rebelse meisjes | Elena Favilli | ROSE Stories | 2 | omnibus edition |
| 143 | Van wie is die staart? | Joukje Akveld | Gottmer | 2,4,5 | non-storybook |
| 151 | Kinderen met een ster | Martine Letterie | Leopold | 4 | omnibus edition |
| 161 | Mooi boek | Joke van Leeuwen | Querido | 4 | omnibus (poems) |
| 169 | Avonturen van Odysseus | Daan Remmerts de Vries | Hoogland & Van Klaveren | 2 | omnibus edition |
| 170 | Stem op de Okapi | Edward van de Vendel | Querido | 2.9 | non-storybook |
| 172 | Doodgewoon | Bette Westera | Gottmer | 2,3,6,9 | omnibus edition |
| 174 | Is er dan niemand boos? | Toon Telligen | Querido | 7.8 | omnibus edition |
| 183 | In volle vaart! | Crushiform | Rubinstein | 2 | non-storybook |
| 184 | Het raadsel van alles wat leeft | Jan Paul Schutten | Gottmer | 3,4,5 | non-storybook |
| 197 | Aan de kant, ik ben je oma niet | Bette Westera | Gottmer | 2.5 | omnibus (poems) |
| 198 | Het leukste abc ter wereld | Tom Schamp | Lannoo | 7 | non-storybook |
| 203 | Nederland | Charlotte Dematons | Lemniscaat | 6 | non-storybook |
| 210 | Vriendjes | Mies van Hout | Lemniscaat | 2 | non-storybook |
| 211 | Een vijver vol inkt | Annie M.G. Schmidt | Querido | 5.6 | omnibus (poems) |
| 215 | Ik en de rovers | Siri Kolu | Gottmer | 4 | book without illustrations |
| 225 | Seizoenen | Blexbolex | Clavis | 5 | non-storybook |
| 228 | Roodkapje was een toffe meid | Marjet Huiberts; Benaissa Linger | Gottmer | 2.4 | omnibus edition |
| 238 | Aadje Piraatje | Marjet Huiberts | Gottmer | 2.4 | omnibus edition |
| 246 | Fluit zoals je bent | Edward van de Vendel | Querido | 4.6 | omnibus (poems) |
| 250 | Driedelig paard | Ted van Lieshout | Leopold | 4.9 | omnibus (poems) |
| 080/253 | Vrolijk | Mies van Hout | Lemniscaat | 10 | non-storybook |
|  |  |  |  |  |  |
| **Total number of excluded books: 35** |  |  |  |  |  |

## Included Awards

|  | **Nederlandse naam** | **English translation** |
| --- | --- | --- |
| 1 | Prentenboek van het Jaar | Picture Book of the Year |
| 2 | Vlag en Wimpels | Flag & Pennants |
| 3 | Gouden Griffel | Golden Slate Pencil |
| 4 | Zilveren Griffel | Silver Slate Pencil |
| 5 | Gouden penseel | Golden Paintbrush |
| 6 | Zilveren penseel | Silver Paintbrush |
| 7 | Gulden palet | Gulden palet |
| 8 | Zilveren palet | Silver palet |
| 9 | Woutertje Pieterse Prijs | Woutertje Pieterse Prize |
| 10 | Kinderboekenwinkelprijs | Children’s Bookshop Prize |

| Appendix B  Codebook | | | |
| --- | --- | --- | --- |
|  |  |  |  |
|  | **Code Name** | **Description** | **Code Label** |
| **1** | book_no | Each sampled book is given a distinct number. | e.g. "001", "002", "003"… |
| **2** | book_title | Title of the book in which the animal is featured. | e.g. "Panda wil een vriendje" |
| **3** | book_year | Year of publication of the book in which the animal is featured. | Year (e.g. "2018") |
| **4** | author_name | Name of the author of the book. | Name |
| **5** | illustrator_name | Name of the illustrator of the book. | Name |
| **6** | publisher_name | Name of the publisher of the book. | Name |
| **7** | publisher_origin | Origin of the publisher of the book in which the animal is featured. | 0=Dutch 1=European 2=Non-European |
| **8** | animal_code | Each distinct animal in the book is given a distinct number; recurrent characters are coded only once, and each animal is coded only once per role (e.g. when three tigers are depicted as minor characters, 'tiger' will be inserted only once; if a tiger is depicted as the main character and another tiger as a minor character, 'tiger' will be inserted two times). | e.g. "1_002" (for the second animal in book 001; "2_003" (for the third animal in book 002) |
| **9** | role | Whether the animal plays a role in the book as the main character (serving as the protagonist), as a supporting character (essential to the storyline yet not a protagonist), or as a minor character (portrayed as part of the scenery/on the background). | 1=main character 2=supporting character 3=minor character |
| **10** | pic_text | Whether the animal is only depicted or also mentioned in text. | 1=image, not in text 2=both text and image |
| **11** | tax_name_text | Most precise name of the animal in the text (at the lowest taxonomic rank possible to determine). When the animal mentioned in the text is clearly a different species than depicted, this will be noted down in the 'notes'. If an animal is only mentioned with a human name (e.g. 'Harry') the animal will be coded with 0 = not mentioned. | Name 0 = not mentioned |
| **12** | tax_rank_text | Taxonomic rank of the most precise name of the animal mentioned in the text. | 0=not mentioned 1=species 2=genus 3=family 4=order 5=class 98=other |
| **13** | tax_name_identified | Everyday name of the depicted animal (at the lowest taxonomic rank possible to determine). If needed, contextual information will be used (text and literature). When the animal is clearly a different species than mentioned in the text, this will be noted down in the 'notes'. | Name |
| **14** | tax_rank_identified | Taxonomic rank of the most precise name of the depicted animal. | 1=species 2=genus 3=family 4=order 5=class 98=other |
| **15** | tax_species | Taxonomic species name of the animal; may be inserted after the initial coding phase. Animals identified above the species level will be coded as 99=unknown. | Name 99=unknown |
| **16** | tax_family | Taxonomic family name of the animal; may be inserted after the initial coding phase. Animals identified above the family level will be coded as 99=unknown. | Name 99=unknown |
| **17** | tax_order | Taxonomic order name of the animal; may be inserted after the initial coding phase. Animals identified above the order level will be coded as 99=unknown. | Name 99=unknown |
| **18** | tax_class | Taxonomic class of the animal; may be inserted after the initial coding phase. We treat dinosaurs as a taxonomic class (to separate them from other reptiles and birds), and we add plesiosaurs, pterosaurs, and sphenacodontids (*Dimetrodon*) to this group. | 1=Mammalia (Mammals) 2=Aves (Birds) 3=Reptilia (Reptiles) 4=Amphibia (Amphibians) 5=Osteichthyes (Ray-finned fish (‘Bony fish’)) 6=Dinosauria (Dinosaurs) 7=Chondrichthyes (Cartilaginous fish) 8=Insecta (Insects) 9=Arachnida (Arachnids) 10=Crustacea (Crustaceans) 11=Gastropoda (Snails, slugs) 12=Cephalopoda (Cephalopods) 13=Bivalvia (Bivalves) 14=Scyphozoa/Cubozoa (Jellyfish) 15=Echinodermata (Echinoderms) 16=Anthozoa (Anthozoans) 20=Other invertebrates 98=Other 99=Unknown |
| **19** | tax_group | Whether the animal is a vertebrate or invertebrate. | 1=vertebrate 2=invertebrate 99=unknown |
| **20** | extinct | Whether the animal is extinct or extant. | 0=extinct (e.g. dinosaur) 1=extant |
| **21** | origin | Whether the animal is native to the Netherlands or exotic. The European rabbit (*Oryctolagus cuniculus*), mallard (*Anas platyrhynchos*), rock pigeon (*Columba livia*), honey bee (*Apis mellifera*), greylag goose (*Anser anser*), and house mouse (*Mus musculus*) will be coded as both domestic and native. | 1=native (for animals specified at species level) 2=exotic (for animals specified at species level (e.g. 'lion)' ánd those at genus/family level for which all species are exotic (e.g. 'penguin' or 'bear') 5=domesticated animal 98=other (e.g. extinct) 99=unknown |
| **22** | type | Type of animal (domestic or non-domestic). A list of domesticated animals from the literature is used. The European rabbit (*Oryctolagus cuniculus*), mallard *(Anas platyrhynchos*), rock pigeon (*Columba livia*), honey bee (*Apis mellifera*), greylag goose (*Anser anser*), and house mouse (*Mus musculus*) will be coded as both domestic and native. | 0=non-domestic/non-pet 1=domestic/pet 98=other (e.g. extinct) 99=unknown |
| **23** | state | Depiction state of the animal (depicted as anthropomorphic or non-anthropomorphic). Similar to Sousa et al. (2017), animals will be coded as anthropomorphic when they show one or more of the following characteristics: wearing clothes or accessories (e.g. jewelry), human behavior (including speech, use of human objects, bipedal walk, and human posture), and human facial features (including facial expressions, blushing cheeks and feminine eyelashes). | 0=non-anthropomorphic 1=anthropomorphic 99=unknown |
| **24** | state_clothing | Whether the animal wears clothing items or human accessories (e.g. jewelry, suitcase). | 0=no 1=yes 99=unknown |
| **25** | state_face | Whether the animal has human facial features (including facial expressions, blushing cheeks and feminine eyelashes). Insects depicted with cartoon eyes (white with a black dot) will be coded as anthropomorphic. | 0=no 1=yes 99=unknown |
| **26** | state_behavior | Whether the animal shows human behavior (including speech, use of human objects, bipedal walk, and human posture). | 0=no 1=yes 99=unknown |
| **27** | cultural_representation | Whether the animal is depicted as a cultural representation (e.g. a toy, painting, statue) or not. | 0=no 1=yes |
| **28** | cultural_representation_type | Type of cultural representation (e.g. a toy, painting, statue). | Name of the type of cultural representation (e.g. 'Toy') |
| **29** | notes | Extra information can be noted here: e.g. whether the animal is a famous cartoon character. |  |

**References mentioned in the codebook**

- Sousa E, Quintino V, Teixeira J, et al. (2017) A portrait of biodiversity in children’s trade books. Society & Animals 25(3): 257–279. DOI: 10.1163/15685306-12341447.

# Appendix C

## Domestic animal species

(Adapted list from [**https://en.wikipedia.org/wiki/List_of_domesticated_animals**](https://en.wikipedia.org/wiki/List_of_domesticated_animals))

Alpaca (*Vicugna pacos*)

Bali cattle (*Bos javanicus domesticus*)

Barbary dove (*Streptopelia roseogrisea risoria*)

Cat (*Felis catus*)

Cattle (*Bos taurus*)

Chicken (*Gallus gallus domesticus*)

Dog (*Canis lupus familiaris*)

Domestic Bactrian camel (*Camelus bactrianus*)

Domestic canary (*Serinus canaria domestica*)

Domestic duck (*Anas platyrhynchos domesticus*)

Domestic goose (*Anser anser domesticus* and *Anser cygnoides domesticus*)

Domestic guinea pig (*Cavia porcellus*)

Domestic guineafowl (*Numida meleagris*)

Domestic mink (*Neovison vison domesticus*)

Domestic muscovy duck (*Cairina moschata domestica*)

Domestic pig (*Sus scrofa domesticus*)

Domestic pigeon (*Columba livia domestica*)

Domestic rabbit (*Oryctolagus cuniculus*)

Domestic silkmoth (*Bombyx mandarina mori*)

Domestic skunk (*Mephitis mephitis*)

Domestic turkey (*Meleagris gallopavo domesticus*)

Donkey (*Equus africanus asinus*)

Dromedary camel (*Camelus dromedarius*)

Fancy mouse and laboratory mouse (*Mus musculus domestica*)

Fancy rat and laboratory rat (*Rattus norvegicus domestica*)

Ferret (*Mustela putorius furo*)

Fuegian dog *(Lycalopex culpaeus*)†

Gayal (*Bos frontalis*)

Goat (*Capra aegagrus hircus*)

Goldfish (*Carassius auratus*)

Horse (*Equus ferus caballus*)

Koi (*Cyprinus rubrofuscus*)

Llama (*Lama glama*)

Sheep (*Ovis aries*)

Siamese fighting fish *(Betta splendens*)

Society finch (*Lonchura striata domestica*)

Water buffalo (*Bubalus bubalis*)

Honey bee (*Apis mellifera*)

Yak (*Bos grunniens*)

Zebu (*Bos taurus indicus*)

# Appendix E

## Top 20 most featured animal orders portrayed in children’s picture books (frequency counts for main, supporting, and minor characters, and total).

| **Order (ordered according to frequency)** | | **Main** | **Supp.** | **Minor** | **Total** |
| --- | --- | --- | --- | --- | --- |
| 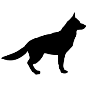 | Carnivora | 46 | 120 | 205 | 16.6% |
| 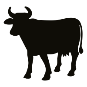 | Cetartiodactyla | 19 | 80 | 121 | 9.8% |
| 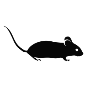 | Rodentia | 11 | 22 | 59 | 4.1% |
| 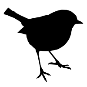 | Passeriformes | 1 | 12 | 77 | 4.0% |
| 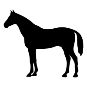 | Perissodactyla | 5 | 25 | 59 | 4.0% |
| 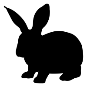 | Lagomorpha | 14 | 19 | 51 | 3.8% |
| 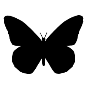 | Lepidoptera | 0 | 8 | 75 | 3.7% |
| 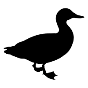 | Anseriformes | 4 | 17 | 44 | 2.9% |
| 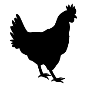 | Galliformes | 3 | 20 | 27 | 2.2% |
| 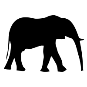 | Proboscidea | 9 | 17 | 22 | 2.1% |
| 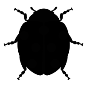 | Coleoptera | 5 | 4 | 32 | 1.8% |
| 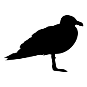 | Charadriiformes | 2 | 4 | 33 | 1.7% |
| 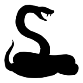 | Squamata | 0 | 6 | 32 | 1.7% |
| 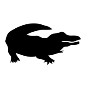 | Crocodilia | 4 | 11 | 19 | 1.5% |
| 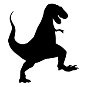 | Saurischia | 0 | 6 | 26 | 1.4% |
| 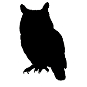 | Strigiformes | 1 | 12 | 17 | 1.3% |
| 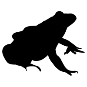 | Anura | 2 | 9 | 17 | 1.3% |
| 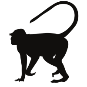 | Primates | 2 | 4 | 19 | 1.1% |
| 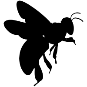 | Hymenoptera | 0 | 10 | 13 | 1.0% |
| 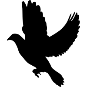 | Columbiformes | 0 | 5 | 14 | 0.8% |
|  | Other | 27 | 133 | 576 | 32.9% |
|  | Total | 155 | 544 | 1538 | 100.0% |

Note: The animal icons in black, dark gray, and light gray represent mammals, birds, and other animals, respectively.

## Top 20 most featured animal species in children’s picture books (frequency counts for main, supporting, and minor characters, and total).

| **Species**  **(ordered according to frequency)** | | **Main** | **Supp.** | **Minor** | **Total** | |
| --- | --- | --- | --- | --- | --- | --- |
| 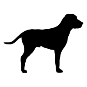 | Dog | 9 | 32 | 64 | 105 | 4.7% |
| 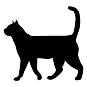 | Cat | 6 | 25 | 49 | 80 | 3.6% |
| 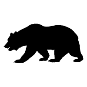 | Brown bear | 12 | 13 | 30 | 55 | 2.5% |
| 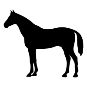 | Horse | 1 | 16 | 38 | 55 | 2.5% |
| 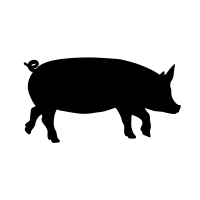 | Pig | 4 | 18 | 22 | 44 | 2.0% |
| 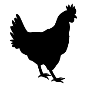 | Chicken | 2 | 15 | 22 | 39 | 1.8% |
| 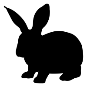 | European rabbit | 8 | 5 | 22 | 35 | 1.6% |
| 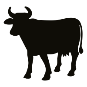 | Cow | 2 | 12 | 14 | 28 | 1.3% |
| 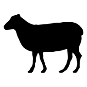 | Sheep | 1 | 15 | 9 | 25 | 1.1% |
| 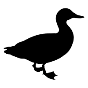 | Mallard | 3 | 9 | 10 | 22 | 1.0% |
| 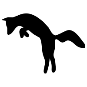 | Red fox | 7 | 3 | 10 | 20 | 0.9% |
| 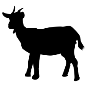 | Goat | 5 | 5 | 10 | 20 | 0.9% |
| 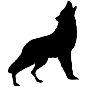 | Wolf | 4 | 8 | 4 | 16 | 0.7% |
| 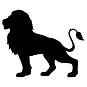 | Lion | 1 | 5 | 9 | 15 | 0.7% |
| 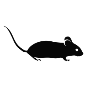 | House mouse | 2 | 5 | 6 | 13 | 0.6% |
| 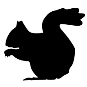 | Red squirrel | 1 | 3 | 9 | 13 | 0.6% |
| 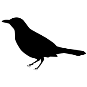 | Common blackbird | 1 | 3 | 8 | 12 | 0.5% |
| 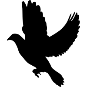 | Rock pigeon | 0 | 3 | 9 | 12 | 0.5% |
| 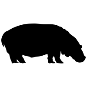 | Hippopotamus | 1 | 6 | 4 | 11 | 0.5% |
| 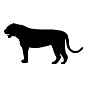 | Tiger | 1 | 6 | 4 | 11 | 0.5% |
|  | Other | 84 | 337 | 1185 | 1606 | 71.8% |
|  | Total | 155 | 544 | 1538 | 2237 | 100.0% |

Note: The animal icons in black represent mammals, those in gray represent birds.

Appendix F

## **Specificity of text references per class.**

|  |  | **Total number of text references** | **Species** | **Genus** | **Family** | **Order** | **Class** | **Other** |
| --- | --- | --- | --- | --- | --- | --- | --- | --- |
| 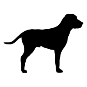 | Mammals | 445 | 61.3% | 5.6% | 27.9% | 5.2% | 0.0% | 0.0% |
| 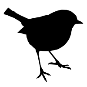 | Birds | 211 | 21.8% | 4.7% | 24.6% | 7.1% | 41.7% | 0.0% |
| 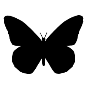 | Insects | 44 | 11.4% | 2.3% | 34.1% | 47.7% | 4.5% | 0.0% |
| 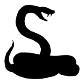 | Reptiles | 34 | 0.0% | 0.0% | 52.9% | 44.1% | 2.9% | 0.0% |
| 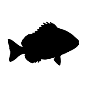 | Bony fish | 30 | 20.0% | 3.3% | 10.0% | 0.0% | 0.0% | 66.7% |
| 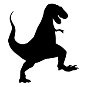 | Dinosaurs | 27 | 0.0% | 14.8% | 0.0% | 0.0% | 85.2% | 0.0% |
| 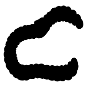 | "Other invertebrates" | 26 | 7.7% | 0.0% | 15.4% | 42.3% | 30.8% | 3.8% |
| 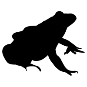 | Amphibians | 11 | 9.1% | 0.0% | 0.0% | 90.9% | 0.0% | 0.0% |
| 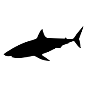 | Cartilaginous fish | 1 | 0.0% | 0.0% | 0.0% | 0.0% | 100.0% | 0.0% |

Note: Percentages indicate the proportion of references at the species, genus, family, order, class, or ‘other’ level.

# Appendix G:

## Prevalence of different types of anthropomorphism in main, supporting, and minor characters.

|  | | **Type of Anthropomorphism** | | | |
| --- | --- | --- | --- | --- | --- |
|  |  | Clothing | Behavior | Facial features | Any |
| **Role** | Main | 34.2% | 81.9% | 91.0% | 96.1% |
|  | Supp. | 17.8% | 40.3% | 55.5% | 63.2% |
|  | Minor | 11.1% | 15.1% | 19.9% | 29.2% |
|  | Total | 14.3% | 25.9% | 33.5% | 42.1% |

## Prevalence of different types of anthropomorphism per class.

|  |  |  | **Type of Anthropomorphism** | | |  |
| --- | --- | --- | --- | --- | --- | --- |
|  | **Class** | **Total** | **Clothing** | **Behavior** | **Facial features** | **Any form** |
| 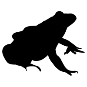 | Amphibians | 30 | 20.0% | 53.3% | 46.7% | 70.0% |
| 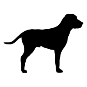 | Mammals | 983 | 22.4% | 37.3% | 46.0% | 57.3% |
| 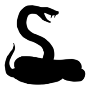 | Reptiles | 90 | 10.0% | 24.4% | 52.2% | 56.7% |
| 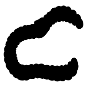 | Other invertebrates | 105 | 4.8% | 14.3% | 41.9% | 43.8% |
| 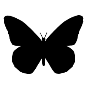 | Insects | 220 | 9.1% | 20.5% | 34.1% | 37.7% |
| 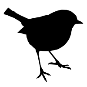 | Birds | 617 | 9.4% | 16.7% | 13.6% | 23.2% |
| 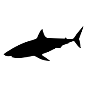 | Cartilaginous fish | 5 | 0.0% | 20.0% | 20.0% | 20.0% |
| 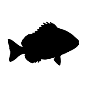 | Bony fish | 128 | 1.6% | 3.9% | 17.2% | 18.8% |
| 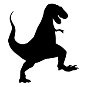 | Dinosaurs | 59 | 1.7% | 8.5% | 16.9% | 16.9% |
|  | Total | 2237 | 321 | 579 | 749 | 942 |

Note: The proportion of animals portrayed anthropomorphically was higher for amphibians than for mammals, but statistical testing revealed that this difference was not significant (χ^2^(1) = 1.931, p=0.165, Cramér’s *V* = 0.044).
